# The $O(\alpha_{s}^{3})$ massive operator matrix elements of $O(n_{f})$ for the structure function $F_{2}(x,Q^{2})$ and transversity

**DOI:** 10.1016/j.nuclphysb.2010.10.021

**Published:** 2011-03-01

**Authors:** J. Ablinger, J. Blümlein, S. Klein, C. Schneider, F. Wißbrock

**Affiliations:** a Research Institute for Symbolic Computation (RISC), Johannes Kepler University, Altenbergerstraße 69, A-4040, Linz, Austria; b Deutsches Elektronen Synchrotron, DESY, Platanenallee 6, D-15738 Zeuthen, Germany; c Institut für Theoretische Teilchenphysik und Kosmologie, RWTH Aachen University, D-52056 Aachen, Germany

## Abstract

The contributions $\propto n_{f}$ to the $O(\alpha_{s}^{3})$ massive operator matrix elements describing the heavy flavor Wilson coefficients in the limit $Q^{2}\gg m^{2}$ are computed for the structure function $F_{2}(x,Q^{2})$ and transversity for general values of the Mellin variable *N*. Here, for two matrix elements, $A_{qq,Q}^{\mathrm{PS}}(N)$ and $A_{qg,Q}(N)$, the complete result is obtained. A first independent computation of the contributions to the 3-loop anomalous dimensions $\gamma_{qg}(N)$, $\gamma_{qq}^{\mathrm{PS}}(N)$, and $\gamma_{qq}^{\mathrm{NS},(\mathrm{TR})}(N)$ is given. In the computation advanced summation technologies for nested sums over products of hypergeometric terms with harmonic sums have been used. For intermediary results generalized harmonic sums occur, while the final results can be expressed by nested harmonic sums only.

## 1 Introduction

The heavy flavor corrections to deep-inelastic structure functions amount to large contributions at lower values of the Bjorken variable *x*. Currently they are known in semi-analytic form to 2-loop (NLO) order [1]. The present accuracy of the deep-inelastic data reaches the order of 1% [2], which requires the next-to-next-to-leading order (NNLO) corrections for precision determinations of both the strong coupling constant $\alpha_{s}(M_{Z}^{2})$ and the parton distribution functions [3], as well as the detailed understanding of the heavy flavor production cross sections in lepton–nucleon scattering [4]. The precise knowledge of these quantities is of central importance for the interpretation of the physics results at the Large Hadron Collider, LHC, [5]. In the region $Q^{2}\gg m^{2}$, with $Q^{2}=-q^{2}$, with *q* the space-like 4-momentum transfer and *m* the heavy quark mass, the power corrections $O({\left(m^{2}/Q^{2}\right)}^{k}),k⩾1$ to the heavy quark structure functions become very small. For the structure function $F_{2}(x,Q^{2})$ the logarithmic and constant contributions are sufficient at the 1%-level to describe the complete result for $Q^{2}/m^{2}\;≳\;10$, a region which does well compare to the deep-inelastic region at HERA in which the twist-2 contributions dominate, cf. [6].^1^ In this limit the Wilson coefficients with $n_{f}$ massless and one massive quark factorize into massive operator matrix elements (OMEs) and the massless Wilson coefficients, as has been shown in Ref. [8]. The former quantities are process independent, while the latter depend on the respective scattering process. The massless Wilson coefficients for the structure function $F_{2}(x,Q^{2})$ are known to 3-loop order, [9].

For fixed Mellin moments *N* a series of moments up to N=10…14, depending on the respective transition, have been calculated for all the OMEs at 3-loop order contributing to the structure function $F_{2}(x,Q^{2})$ and those needed to establish a variable flavor scheme description at $O(\alpha_{s}^{3})$ in Ref. [10].^2^ There also the complete renormalization of the matrix elements has been derived. In this computation the massive OMEs for given total spin *N* were mapped onto massive tadpoles which were computed using MATAD, [12]. For general values of *N* the 2-loop OMEs, up to O(ε), have been calculated in Refs. [13], [14], [15]. All the logarithmic contributions to the massive OMEs are known [16], [17] for general values of *N*. They depend on the 3-loop anomalous dimensions [18], [19]. For the structure function $F_{L}(x,Q^{2})$ the asymptotic heavy flavor Wilson coefficients at $O(\alpha_{s}^{3})$ were calculated in [20]. They become, however, effective only at much higher scales of $Q^{2}$ compared to the case of $F_{2}(x,Q^{2})$.

In the present paper the $O(\alpha_{s}^{3})$ contributions $\propto n_{F}T_{F}^{2}C_{F,A}$ are computed for all massive operator matrix elements contributing to the structure function $F_{2}(x,Q^{2})$ at general values of the Mellin variable *N* in the fixed flavor number scheme, as well as the corresponding contributions to transversity. This scheme has to be considered as the genuine scheme in quantum field theoretic calculations since the initial states, the twist-2 *massless* partons can, at least to a good approximation, be considered as LSZ-states. This is not the case for heavy quark states, which have a finite lifetime.^3^ For two OMEs, $A_{qq,Q}^{\mathrm{PS}}(N)$ and $A_{qg,Q}(N)$, the complete result is obtained. In the present computation the Feynman parameter integrals are computed directly. They can be represented in terms of generalized hypergeometric functions [22] and sums thereof prior the expansion in the dimensional variable ε=D−4, cf. [23], [24]. Finally, they are represented in terms of nested sums over products of hypergeometric terms and harmonic sums, which can be calculated using modern summation techniques [25], [26] that are based on a refined difference field of [27] and that generalize the summation paradigms presented in [28] to multi-summation. During this computation the results can be expressed in terms of nested harmonic sums [29], [30]. In intermediary steps of the calculation generalized harmonic sums, [31], [32], cf. also [33], appear which finally cancel.

The paper is organized as follows. In Section 2 we summarize the basic formalism. The results for the constant part of the $O(\alpha_{s}^{3})$ $n_{f}$-contributions to the massive OMEs ${\hat{A}}_{Qg}(N)$, ${\hat{A}}_{Qq}^{\mathrm{PS}}(N)$ ${\hat{A}}_{qq,Q}^{\mathrm{NS}}(N)$, ${\hat{A}}_{qg,Q}(N)$, ${\hat{A}}_{qq,Q}^{\mathrm{PS}}(N)$, and ${\hat{A}}_{qq,Q}^{\mathrm{NS},\mathrm{TR}}(N)$, cf. [10], [11], are presented in Section 3. The single pole terms in *ε* allow to derive the terms $\propto n_{f}$ of the 3-loop anomalous dimensions for general values of *N*. They are compared to the results in Refs. [18], [19], [34] and are obtained in a first independent calculation for $\gamma_{qg}(N)$, $\gamma_{qq}^{\mathrm{PS}}(N)$, $\gamma_{qq}^{\mathrm{NS},\mathrm{TR}}(N)$, in Section 4. Section 5 contains the conclusions. Some technical details of the calculation are given in Appendix A.

## 2 The heavy flavor Wilson coefficients in the asymptotic region

The heavy flavor contributions to the structure functions $F_{\left(2,L\right)}(x,Q^{2})$ with $n_{f}$ massless and one heavy flavor are given by, [10]:

(1) $$F_{\left(2,L\right)}^{Q\bar{Q}}\left(x,n_{f}+1,Q^{2},m^{2}\right)=\sum_{k=1}^{n_{f}}e_{k}^{2}\left\{L_{q,(2,L)}^{\mathrm{NS}}\left(x,n_{f}+1,\frac{Q^{2}}{m^{2}},\frac{m^{2}}{\mu^{2}}\right)⊗\left[f_{k}\left(x,\mu^{2},n_{f}\right)+f_{\bar{k}}\left(x,\mu^{2},n_{f}\right)\right]+\frac{1}{n_{f}}\left[L_{q,(2,L)}^{\mathrm{PS}}\left(x,n_{f}+1,\frac{Q^{2}}{m^{2}},\frac{m^{2}}{\mu^{2}}\right)⊗\Sigma \left(x,\mu^{2},n_{f}\right)+L_{g,(2,L)}^{S}\left(x,n_{f}+1,\frac{Q^{2}}{m^{2}},\frac{m^{2}}{\mu^{2}}\right)⊗G\left(x,\mu^{2},n_{f}\right)\right]\right\}+e_{Q}^{2}\left[H_{q,(2,L)}^{\mathrm{PS}}\left(x,n_{f}+1,\frac{Q^{2}}{m^{2}},\frac{m^{2}}{\mu^{2}}\right)⊗\Sigma \left(x,\mu^{2},n_{f}\right)+H_{g,(2,L)}^{S}\left(x,n_{f}+1,\frac{Q^{2}}{m^{2}},\frac{m^{2}}{\mu^{2}}\right)⊗G\left(x,\mu^{2},n_{f}\right)\right].$$

The different Wilson coefficients are denoted by $L_{i},H_{i}$ in case the photon couples to a light (*L*) or the heavy (*H*) quark, for the flavor non-singlet (NS), pure-singlet (PS), and singlet (S) cases. Here, ⊗ is the Mellin convolution,

(2) $$[A⊗B](x)=\int_{0}^{1}\int_{0}^{1}dx_{1}\;dx_{2}\;\delta (x-x_{1}x_{2})A(x_{1})B(x_{2}),$$

with boundaries for the Wilson coefficients $\left[x(1+4m^{2}/Q^{2}),1\right]$, $e_{k}$ the light and $e_{Q}$ the heavy quark charges. $\mu^{2}$ denotes the factorization scale, and $f_{k},f_{\bar{k}},\Sigma$ and *G* are the quark, antiquark, flavor singlet and gluon momentum distribution functions, with

(3) $$\Sigma \left(x,\mu^{2},n_{f}\right)=\sum_{k=1}^{n_{f}}\left[f_{k}\left(x,\mu^{2},n_{f}\right)+f_{\bar{k}}\left(x,\mu^{2},n_{f}\right)\right].$$

For $Q^{2}\gg m^{2}$ the massive Wilson coefficients can be expressed in terms of the renormalized massive OMEs $A_{ij}$ and the massless Wilson coefficients $C_{j}$. To $O(a_{s}^{3})$ they read ($a_{s}=\alpha_{s}/(4\pi )$), cf. [10]:

$$L_{q,(2,L)}^{\mathrm{NS}}(n_{f}+1)=a_{s}^{2}\left[A_{qq,Q}^{(2),\mathrm{NS}}(n_{f}+1)\delta_{2}+{\hat{C}}_{q,(2,L)}^{(2),\mathrm{NS}}(n_{f})\right]+a_{s}^{3}\left[A_{qq,Q}^{(3),\mathrm{NS}}(n_{f}+1)\delta_{2}+A_{qq,Q}^{(2),\mathrm{NS}}(n_{f}+1)C_{q,(2,L)}^{(1),\mathrm{NS}}(n_{f}+1)+{\hat{C}}_{q,(2,L)}^{(3),\mathrm{NS}}(n_{f})\right],$$

$$L_{q,(2,L)}^{\mathrm{PS}}(n_{f}+1)=a_{s}^{3}\left[A_{qq,Q}^{(3),\mathrm{PS}}(n_{f}+1)\delta_{2}+A_{gq,Q}^{\left(2\right)}(n_{f})n_{f}{\tilde{C}}_{g,(2,L)}^{\left(1\right)}(n_{f}+1)+n_{f}{\hat{\tilde{C}}}_{q,(2,L)}^{(3),\mathrm{PS}}(n_{f})\right],$$

(4) $$L_{g,(2,L)}^{S}(n_{f}+1)=a_{s}^{2}A_{gg,Q}^{\left(1\right)}(n_{f}+1)n_{f}{\tilde{C}}_{g,(2,L)}^{\left(1\right)}(n_{f}+1)+a_{s}^{3}\left[A_{qg,Q}^{\left(3\right)}(n_{f}+1)\delta_{2}+A_{gg,Q}^{\left(1\right)}(n_{f}+1)n_{f}{\tilde{C}}_{g,(2,L)}^{\left(2\right)}(n_{f}+1)+A_{gg,Q}^{\left(2\right)}(n_{f}+1)n_{f}{\tilde{C}}_{g,(2,L)}^{\left(1\right)}(n_{f}+1)+A_{Qg}^{\left(1\right)}(n_{f}+1)n_{f}{\tilde{C}}_{q,(2,L)}^{(2),\mathrm{PS}}(n_{f}+1)+n_{f}{\hat{\tilde{C}}}_{g,(2,L)}^{\left(3\right)}(n_{f})\right],$$

$$H_{q,(2,L)}^{\mathrm{PS}}(n_{f}+1)=a_{s}^{2}\left[A_{Qq}^{(2),\mathrm{PS}}(n_{f}+1)\delta_{2}+{\tilde{C}}_{q,(2,L)}^{(2),\mathrm{PS}}(n_{f}+1)\right]+a_{s}^{3}\left[A_{Qq}^{(3),\mathrm{PS}}(n_{f}+1)\delta_{2}+{\tilde{C}}_{q,(2,L)}^{(3),\mathrm{PS}}(n_{f}+1)+A_{gq,Q}^{\left(2\right)}(n_{f}+1){\tilde{C}}_{g,(2,L)}^{\left(1\right)}(n_{f}+1)+A_{Qq}^{(2),\mathrm{PS}}(n_{f}+1)C_{q,(2,L)}^{(1),\mathrm{NS}}(n_{f}+1)\right],$$

$$H_{g,(2,L)}^{S}(n_{f}+1)=a_{s}\left[A_{Qg}^{\left(1\right)}(n_{f}+1)\delta_{2}+{\tilde{C}}_{g,(2,L)}^{\left(1\right)}(n_{f}+1)\right]+a_{s}^{2}\left[A_{Qg}^{\left(2\right)}(n_{f}+1)\delta_{2}+A_{Qg}^{\left(1\right)}(n_{f}+1)C_{q,(2,L)}^{(1),\mathrm{NS}}(n_{f}+1)+A_{gg,Q}^{\left(1\right)}(n_{f}+1){\tilde{C}}_{g,(2,L)}^{\left(1\right)}(n_{f}+1)+{\tilde{C}}_{g,(2,L)}^{\left(2\right)}(n_{f}+1)\right]+a_{s}^{3}\left[A_{Qg}^{\left(3\right)}(n_{f}+1)\delta_{2}+A_{Qg}^{\left(2\right)}(n_{f}+1)C_{q,(2,L)}^{(1),\mathrm{NS}}(n_{f}+1)+A_{gg,Q}^{\left(2\right)}(n_{f}+1){\tilde{C}}_{g,(2,L)}^{\left(1\right)}(n_{f}+1)+A_{Qg}^{\left(1\right)}(n_{f}+1)\left\{C_{q,(2,L)}^{(2),\mathrm{NS}}(n_{f}+1)+{\tilde{C}}_{q,(2,L)}^{(2),\mathrm{PS}}(n_{f}+1)\right\}+A_{gg,Q}^{\left(1\right)}(n_{f}+1){\tilde{C}}_{g,(2,L)}^{\left(2\right)}(n_{f}+1)+{\tilde{C}}_{g,(2,L)}^{\left(3\right)}(n_{f}+1)\right],$$

with $\delta_{2}=0(1)$ for $F_{L}(F_{2})$ and $\hat{f}(n_{f})=f(n_{f}+1)-f(n_{f}),\tilde{f}(n_{f})=f(n_{f})/n_{f}$.

The renormalized massive OMEs depend on the ratio $m^{2}/\mu^{2}$, while the scale ratio in the massless Wilson coefficients is $\mu^{2}/Q^{2}$. The latter are pure functions of the momentum fraction *z*, or the Mellin variable *N*, if one sets $\mu^{2}=Q^{2}$. The mass dependence of the heavy flavor Wilson coefficients in the asymptotic region derives from the unrenormalized massive OMEs

(5) $${\hat{A}}_{ij}^{\left(3\right)}(\varepsilon )=\frac{1}{\varepsilon^{3}}{\hat{a}}_{ij}^{(3),3}+\frac{1}{\varepsilon^{2}}{\hat{a}}_{ij}^{(3),2}+\frac{1}{\varepsilon }{\hat{a}}_{ij}^{(3),1}+{\hat{a}}_{ij}^{(3),0},$$

applying mass, coupling constant, and operator-renormalization, as well as mass factorization, cf. Ref. [10].

The renormalized massive OMEs obey then the general structure

(6) $$A_{ij}^{\left(3\right)}\left(\frac{m^{2}}{Q^{2}}\right)=a_{ij}^{(3),3}\ln^{3}\left(\frac{m^{2}}{Q^{2}}\right)+a_{ij}^{(3),2}\ln^{2}\left(\frac{m^{2}}{Q^{2}}\right)+a_{ij}^{(3),1}\ln\left(\frac{m^{2}}{Q^{2}}\right)+a_{ij}^{(3),0}.$$

The subsequent calculations will be performed in the $\bar{\mathrm{MS}}$ scheme. For other scheme choices see Ref. [10]. Therefore the strong coupling constant is obtained as the *perturbative* solution of the equation

(7) $$\frac{da_{s}(\mu^{2})}{d\ln(\mu^{2})}=-\sum_{l=0}^{\infty }\beta_{l}a_{s}^{l+2}\left(\mu^{2}\right)$$

to 3-loop order, where $\beta_{k}$ are the expansion coefficients of the QCD *β*-function and $\mu^{2}$ denotes the renormalization scale. For simplicity we identify the factorization and renormalization scales in the following.

## 3 The massive operator matrix elements

The operator matrix elements $\propto n_{f}$ for both $F_{2}(x,Q^{2})$ and transversity are obtained by the massive two-loop graphs inserting a further massless fermion line and new planar three-loop topologies, cf. [35], [36], as well as 3-loop graphs containing bubble topologies with operator insertions linked either linked to massive or massless fermion lines, cf. [10]. The calculation was carried out in Feynman-gauge^4^ using FORM [37] and MAPLE-codes, and applied the package color [38] for the color algebra. As in earlier cases [15] we computed the Feynman parameter-integrals directly, without applying the integrating-by-parts method [39]. The corresponding integrals can be mapped onto sums over generalized hypergeometric functions prior the *ε*-expansion, which allow to obtain the Laurent series in *ε*. Finally, up to three-fold nested sums over hypergeometric expressions, equipped with harmonic sums, have to be performed, for which the package Sigma [25], constructing difference and product fields, was applied and extended. Some details of the computation are presented in Appendix A.

The massive OMEs $A_{ij}^{\left(k\right)}(N)$ are finally obtained as rational functions of the Mellin variable *N*, multiple zeta values [40], and nested harmonic sums [29], [30]. The latter are defined recursively by

(8) $$S_{b,\vec{a}}(N)=\sum_{k=1}^{N}\frac{(\mathrm{sign}{\left(b)\right)}^{k}}{k^{\left|b\right|}}S_{\vec{a}}(k),\;S_{\emptyset }(N)=1.$$

As a short-hand notation we use $S_{\vec{a}}(N)\equiv S_{\vec{a}}$. In representing the results, the algebraic relations of the nested harmonic sums [41] are applied. In the following we present the constant contributions to the unrenormalized OMEs (5) as genuine quantities, to allow for different scheme choices, cf. Ref. [10].

### 3.1 Operator matrix elements contributing to $F_{2}(x,Q^{2})$

The $O(n_{f})$ contribution to the unrenormalized OME ${\hat{A}}_{Qg}^{\left(3\right)}(\varepsilon ,N)$, ${\hat{a}}_{Qg}^{(3),0}$, reads:

(9) $${\hat{a}}_{Qg}^{(3),0}=n_{f}T_{F}^{2}C_{A}\left\{\frac{16(N^{2}+N+2)}{27N(N+1)(N+2)}\left[108S_{-2,1,1}-78S_{2,1,1}-90S_{-3,1}+72S_{2,-2}-6S_{3,1}-108S_{-2,1}S_{1}+42S_{2,1}S_{1}-6S_{-4}+90S_{-3}S_{1}+118S_{3}S_{1}+120S_{4}+18S_{-2}S_{2}+54S_{-2}S_{1}^{2}+33S_{2}S_{1}^{2}+15S_{2}^{2}+2S_{1}^{4}+18S_{-2}\zeta_{2}+9S_{2}\zeta_{2}+9S_{1}^{2}\zeta_{2}-42S_{1}\zeta_{3}\right]+32\frac{5N^{4}+14N^{3}+53N^{2}+82N+20}{27N{\left(N+1\right)}^{2}{\left(N+2\right)}^{2}}[6S_{-2,1}-5S_{-3}-6S_{-2}S_{1}]-\frac{64(5N^{4}+11N^{3}+50N^{2}+85N+20)}{27N{\left(N+1\right)}^{2}{\left(N+2\right)}^{2}}S_{2,1}-\frac{16(40N^{4}+151N^{3}+544N^{2}+779N+214)}{27N{\left(N+1\right)}^{2}{\left(N+2\right)}^{2}}S_{2}S_{1}-\frac{32(65N^{6}+429N^{5}+1155N^{4}+725N^{3}+370N^{2}+496N+648)}{81(N-1)N^{2}{\left(N+1\right)}^{2}{\left(N+2\right)}^{2}}S_{3}-\frac{16(20N^{4}+107N^{3}+344N^{2}+439N+134)}{81N{\left(N+1\right)}^{2}{\left(N+2\right)}^{2}}S_{1}^{3}+\frac{Q_{1}(N)}{81(N-1)N^{3}{\left(N+1\right)}^{3}{\left(N+2\right)}^{3}}S_{2}+\frac{32(47N^{6}+278N^{5}+1257N^{4}+2552N^{3}+1794N^{2}+284N+448)}{81N{\left(N+1\right)}^{3}{\left(N+2\right)}^{3}}S_{-2}+\frac{8(22N^{6}+271N^{5}+2355N^{4}+6430N^{3}+6816N^{2}+3172N+1256)}{81N{\left(N+1\right)}^{3}{\left(N+2\right)}^{3}}S_{1}^{2}+\frac{Q_{2}(N)}{243(N-1)N^{2}{\left(N+1\right)}^{4}{\left(N+2\right)}^{4}}S_{1}+\frac{448(N^{2}+N+1)(N^{2}+N+2)}{9(N-1)N^{2}{\left(N+1\right)}^{2}{\left(N+2\right)}^{2}}\zeta_{3}-\frac{16(5N^{4}+20N^{3}+59N^{2}+76N+20)}{9N{\left(N+1\right)}^{2}{\left(N+2\right)}^{2}}S_{1}\zeta_{2}-\frac{Q_{3}(N)}{9(N-1)N^{3}{\left(N+1\right)}^{3}{\left(N+2\right)}^{3}}\zeta_{2}-\frac{Q_{4}(N)}{243(N-1)N^{5}{\left(N+1\right)}^{5}{\left(N+2\right)}^{5}}\right\}+n_{f}T_{F}^{2}C_{F}\left\{\frac{16(N^{2}+N+2)}{27N(N+1)(N+2)}\left[144S_{2,1,1}-72S_{3,1}-72S_{2,1}S_{1}+48S_{4}-16S_{3}S_{1}-24S_{2}^{2}-12S_{2}S_{1}^{2}-2S_{1}^{4}-9S_{1}^{2}\zeta_{2}+42S_{1}\zeta_{3}\right]+32\frac{10N^{3}+49N^{2}+83N+24}{81N^{2}(N+1)(N+2)}\left[3S_{2}S_{1}+S_{1}^{3}\right]-\frac{128(N^{2}-3N-2)}{3N^{2}(N+1)(N+2)}S_{2,1}-\frac{Q_{5}(N)}{81(N-1)N^{3}{\left(N+1\right)}^{3}{\left(N+2\right)}^{2}}S_{3}+\frac{Q_{6}(N)}{27(N-1)N^{4}{\left(N+1\right)}^{4}{\left(N+2\right)}^{3}}S_{2}-\frac{32(10N^{4}+185N^{3}+789N^{2}+521N+141)}{81N^{2}{\left(N+1\right)}^{2}(N+2)}S_{1}^{2}-\frac{16(230N^{5}-924N^{4}-5165N^{3}-7454N^{2}-10217N-2670)}{243N^{2}{\left(N+1\right)}^{3}(N+2)}S_{1}+\frac{16(5N^{3}+11N^{2}+28N+12)}{9N^{2}(N+1)(N+2)}S_{1}\zeta_{2}-\frac{Q_{7}(N)}{9(N-1)N^{3}{\left(N+1\right)}^{3}{\left(N+2\right)}^{2}}\zeta_{3}+\frac{Q_{8}(N)}{9(N-1)N^{4}{\left(N+1\right)}^{4}{\left(N+2\right)}^{3}}\zeta_{2}+\frac{Q_{9}(N)}{243(N-1)N^{6}{\left(N+1\right)}^{6}{\left(N+2\right)}^{5}}\right\},$$

with the polynomials

(10) $$Q_{1}(N)=32N^{9}-936N^{8}+6448N^{7}+55208N^{6}+126160N^{5}+61760N^{4}-53152N^{3}-25024N^{2}-32256N-13824,$$

(11) $$Q_{2}(N)=7856N^{10}+84672N^{9}+377648N^{8}+985568N^{7}+1395456N^{6}+470688N^{5}-1183712N^{4}-1180224N^{3}-182528N^{2}-42752N+13824,$$

(12) $$Q_{3}(N)=60N^{9}+360N^{8}+584N^{7}-128N^{6}-2004N^{5}-2440N^{4}-976N^{3}-192N^{2}+896N+384,$$

(13) $$Q_{4}(N)=28776N^{15}+356112N^{14}+1896088N^{13}+5538320N^{12}+9112264N^{11}+6793968N^{10}-3019528N^{9}-11879520N^{8}-11673088N^{7}-6450992N^{6}-3726976N^{5}-2248128N^{4}-183296N^{3}+268032N^{2}+147456N+27648,$$

(14) $$Q_{5}(N)=464N^{8}-15616N^{7}-38112N^{6}+27776N^{5}+146064N^{4}+119552N^{3}+109312N^{2}+86016N+62208,$$

(15) $$Q_{6}(N)=456N^{11}+4376N^{10}+11328N^{9}-3184N^{8}-54552N^{7}-111720N^{6}-155376N^{5}-251072N^{4}-312192N^{3}-222464N^{2}-135936N-41472,$$

(16) $$Q_{7}(N)=168N^{8}+672N^{7}+784N^{6}-3192N^{4}-5600N^{3}-7168N^{2}-4480N-2688,$$

(17) $$Q_{8}(N)=90N^{11}+630N^{10}+1592N^{9}+1260N^{8}-1934N^{7}-8218N^{6}-15524N^{5}-23944N^{4}-26752N^{3}-18400N^{2}-11328N-3456,$$

(18) $$Q_{9}(N)=15777N^{17}+186525N^{16}+879391N^{15}+1874085N^{14}+575913N^{13}-5568833N^{12}-10465411N^{11}-2970289N^{10}+11884298N^{9}+12640320N^{8}-10343664N^{7}-40750480N^{6}-55711424N^{5}-53947712N^{4}-42534912N^{3}-23256576N^{2}-7865856N-1244160,$$

and

(19) $$\zeta_{k}=\sum_{l=1}^{\infty }\frac{1}{l^{k}},\;k\in N,\;k⩾2$$

denotes the Riemann *ζ*-function.

The corresponding contribution to the pure singlet OME ${\hat{A}}_{Qq}^{\mathrm{PS},(3)}(\varepsilon ,N)$ is given by

(20) $${\hat{a}}_{Qq}^{\mathrm{PS},(3),0}=\frac{n_{f}T_{F}^{2}C_{F}}{N^{2}{\left(1+N\right)}^{2}(2+N)(N-1)}\left\{{\left(N^{2}+N+2\right)}^{2}\left(-\frac{1760}{27}S_{3}-\frac{208}{9}S_{2}S_{1}-\frac{16}{27}S_{1}^{3}-\frac{16}{3}S_{1}\zeta_{2}+\frac{224}{9}\zeta_{3}\right)+\frac{Q_{10}(N)}{N(1+N)(2+N)}\left[\frac{208}{27}S_{2}+\frac{16}{27}S_{1}^{2}+\frac{16}{9}\zeta_{2}\right]-\frac{32}{81}\frac{Q_{11}(N)}{N^{2}{\left(1+N\right)}^{2}{\left(2+N\right)}^{2}}S_{1}+\frac{32}{243}\frac{Q_{12}(N)}{N^{3}{\left(1+N\right)}^{3}{\left(2+N\right)}^{3}}\right\},$$

with

(21) $$Q_{10}(N)=8N^{7}+37N^{6}+68N^{5}-11N^{4}-86N^{3}-56N^{2}-104N-48,$$

(22) $$Q_{11}(N)=25N^{10}+176N^{9}+417N^{8}+30N^{7}-20N^{6}+1848N^{5}+2244N^{4}+1648N^{3}+3040N^{2}+2112N+576,$$

(23) $$Q_{12}(N)=158N^{13}+1663N^{12}+7714N^{11}+23003N^{10}+56186N^{9}+89880N^{8}+59452N^{7}-8896N^{6}-12856N^{5}-24944N^{4}-84608N^{3}-77952N^{2}-35712N-6912,$$

The second pure-singlet operator matrix element is ${\hat{A}}_{qq,Q}^{\mathrm{PS},(3)}(\varepsilon ,N)$. Its constant term reads:

(24) $${\hat{a}}_{qq,Q}^{\mathrm{PS},(3),0}=\frac{n_{f}T_{F}^{2}C_{F}}{N^{2}(N-1)(2+N){\left(1+N\right)}^{2}}\left\{{\left(N^{2}+N+2\right)}^{2}\left(\frac{256}{27}S_{3}+\frac{128}{9}S_{2}S_{1}+\frac{128}{27}S_{1}^{3}+\frac{32}{3}S_{1}\zeta_{2}+\frac{224}{9}\zeta_{3}\right)-\frac{Q_{13}(N)}{N(2+N)(1+N)}\left[\frac{64}{27}S_{2}+\frac{64}{27}S_{1}^{2}+\frac{16}{9}\zeta_{2}\right]+\frac{64}{81}\frac{Q_{14}(N)}{N^{2}{\left(2+N\right)}^{2}{\left(1+N\right)}^{2}}S_{1}-\frac{32}{243}\frac{Q_{15}(N)}{N^{3}{\left(2+N\right)}^{3}{\left(1+N\right)}^{3}}\right\},$$

with

(25) $$Q_{13}(N)=16N^{7}+74N^{6}+181N^{5}+266N^{4}+269N^{3}+230N^{2}+44N-24,$$

(26) $$Q_{14}(N)=181N^{10}+1352N^{9}+4737N^{8}+10101N^{7}+14923N^{6}+17085N^{5}+14133N^{4}+5944N^{3}+568N^{2}-48N+144,$$

(27) $$Q_{15}(N)=2074N^{13}+21728N^{12}+105173N^{11}+311482N^{10}+636490N^{9}+966828N^{8}+1126568N^{7}+968818N^{6}+550813N^{5}+169250N^{4}+12104N^{3}-3408N^{2}-1008N-864.$$

The constant term of the unrenormalized flavor non-singlet operator matrix element ${\hat{A}}_{qq,Q}^{\mathrm{NS},(3)}(\varepsilon ,N)$ is given by:

(28) $${\hat{a}}_{qq,Q}^{\mathrm{NS},(3),0}=n_{f}T_{F}^{2}C_{F}\left\{\frac{64}{27}S_{4}+\frac{448}{27}\zeta_{3}S_{1}+\frac{32}{9}\zeta_{2}S_{2}-\frac{320}{81}S_{3}-\frac{160}{27}\zeta_{2}S_{1}-\frac{112}{27}\frac{3N^{2}+3N+2}{(1+N)N}\zeta_{3}+\frac{640}{27}S_{2}+\frac{4}{27}\frac{3N^{4}+6N^{3}+47N^{2}+20N-12}{{\left(1+N\right)}^{2}N^{2}}\zeta_{2}-\frac{55552}{729}S_{1}+\frac{2}{729}\frac{Q_{16}(N)}{{\left(1+N\right)}^{4}N^{4}}\right\},$$

where

(29) $$Q_{16}(N)=11751N^{8}+47004N^{7}+93754N^{6}+104364N^{5}+55287N^{4}+6256N^{3}-2448N^{2}-144N-432.$$

Finally, the constant contribution to ${\hat{A}}_{qg,Q}^{\left(3\right)}(\varepsilon ,N)$ at $O(n_{f})$ is:

(30) $${\hat{a}}_{qg,Q}^{(3),0}=\frac{n_{f}T_{F}^{2}}{N(N+1)(N+2)}\left\{C_{F}\left[\left(N^{2}+N+2\right)\left(\frac{4}{27}S_{1}^{4}+\frac{8}{3}\zeta_{2}S_{1}^{2}+\frac{8}{9}S_{2}S_{1}^{2}+\frac{224}{9}\zeta_{3}S_{1}+\frac{32}{27}S_{3}S_{1}+\frac{4}{9}S_{2}^{2}+8\zeta_{2}S_{2}+\frac{40}{9}S_{4}-\frac{56Q_{17}(N)}{9(N-1)N^{2}{\left(N+1\right)}^{2}(N+2)}\zeta_{3}\right)-\frac{16(10N^{3}+13N^{2}+29N+6)S_{1}^{3}}{81N}+\frac{8(215N^{4}+481N^{3}+930N^{2}+748N+120)}{81N(N+1)}S_{1}^{2}-\frac{16(10N^{3}+13N^{2}+29N+6)}{27N}(3\zeta_{2}S_{1}+S_{2}S_{1})-\frac{32(40N^{3}+61N^{2}+89N+6)}{81N}S_{3}+\frac{8(221N^{4}+515N^{3}+814N^{2}+548N+40)}{27N(N+1)}S_{2}+\frac{4Q_{18}(N)}{9(N-1)N^{3}{\left(N+1\right)}^{3}{\left(N+2\right)}^{2}}\zeta_{2}-\frac{16Q_{19}(N)}{243N{\left(N+1\right)}^{2}}S_{1}+\frac{8Q_{20}(N)}{243(N-1)N^{5}{\left(N+1\right)}^{5}{\left(N+2\right)}^{4}}\right]+C_{A}\left[\left(N^{2}+N+2\right)\left(-\frac{4}{27}S_{1}^{4}-\frac{8}{3}\zeta_{2}S_{1}^{2}+\frac{8}{9}S_{2}S_{1}^{2}-\frac{56}{9}S_{4}-\frac{128}{9}S_{3,1}+\frac{64}{9}S_{2,1,1}+\frac{160}{27}S_{3}S_{1}-\frac{64}{9}S_{2,1}S_{1}-\frac{4}{9}S_{2}^{2}-\frac{128}{9}S_{-4}-\frac{224}{9}\zeta_{3}S_{1}-\frac{16}{3}\zeta_{2}S_{-2}-\frac{8}{3}\zeta_{2}S_{2}+\frac{448(N^{2}+N+1)}{9(N-1)N(N+1)(N+2)}\zeta_{3}\right)+\frac{32(5N^{4}+20N^{3}+41N^{2}+49N+20)}{81(N+1)(N+2)}\left(9\zeta_{2}S_{1}-3S_{2}S_{1}+12S_{2,1}+S_{1}^{3}\right)+\frac{64(5N^{4}+38N^{3}+59N^{2}+31N+20)}{81(N+1)(N+2)}S_{3}+\frac{128}{27}\left(5N^{2}+8N+10\right)S_{-3}-\frac{8Q_{21}(N)}{81{\left(N+1\right)}^{2}{\left(N+2\right)}^{2}}S_{1}^{2}+\frac{8Q_{22}(N)}{9(N-1)N^{2}{\left(N+1\right)}^{2}{\left(N+2\right)}^{2}}\zeta_{2}-\frac{32(121N^{3}+293N^{2}+414N+224)}{81(N+1)}S_{-2}-\frac{8Q_{23}(N)}{81{\left(N+1\right)}^{2}{\left(N+2\right)}^{2}}S_{2}+\frac{16Q_{24}(N)}{243(N-1)N{\left(N+1\right)}^{3}{\left(N+2\right)}^{3}}S_{1}+\frac{16Q_{25}(N)}{243(N-1)N^{4}{\left(N+1\right)}^{4}{\left(N+2\right)}^{4}}\right]\right\},$$

with

(31) $$Q_{17}(N)=3N^{6}+9N^{5}-N^{4}-17N^{3}-38N^{2}-28N-24,$$

(32) $$Q_{18}(N)=18N^{11}+126N^{10}+365N^{9}+630N^{8}+652N^{7}+626N^{6}+1309N^{5}+3170N^{4}+4736N^{3}+3584N^{2}+2352N+864,$$

(33) $$Q_{19}(N)=2507N^{5}+8076N^{4}+16120N^{3}+18997N^{2}+9898N+1344,$$

(34) $$Q_{20}(N)=2322N^{17}+30186N^{16}+177047N^{15}+627060N^{14}+1509207N^{13}+2623160N^{12}+3436402N^{11}+3728602N^{10}+4151281N^{9}+5013306N^{8}+5011065N^{7}+3770902N^{6}+3291500N^{5}+3951272N^{4}+3797616N^{3}+2319264N^{2}+862272N+155520,$$

(35) $$Q_{21}(N)=206N^{6}+1361N^{5}+4134N^{4}+7577N^{3}+8394N^{2}+4868N+1144,$$

(36) $$Q_{22}(N)=6N^{9}+36N^{8}+11N^{7}-257N^{6}-825N^{5}-1375N^{4}-1396N^{3}-984N^{2}-352N-48,$$

(37) $$Q_{23}(N)=332N^{6}+2537N^{5}+7848N^{4}+13145N^{3}+13122N^{2}+7412N+1720,$$

(38) $$Q_{24}(N)=2228N^{10}+19197N^{9}+72518N^{8}+155774N^{7}+193362N^{6}+94317N^{5}-87644N^{4}-163656N^{3}-91040N^{2}-11888N+3456,$$

(39) $$Q_{25}(N)=2040N^{15}+24480N^{14}+116165N^{13}+254533N^{12}+78119N^{11}-1089300N^{10}-3414794N^{9}-5743128N^{8}-6358562N^{7}-4824553N^{6}-2448740N^{5}-783540N^{4}-213184N^{3}-155568N^{2}-97344N-22464.$$

We compared ${\hat{a}}_{Qg}^{(3),0}(N)$, ${\hat{a}}_{Qq}^{\mathrm{PS},(3),0}(N)$, ${\hat{a}}_{qq,Q}^{\mathrm{PS},(3),0}(N)$, ${\hat{a}}_{qq,Q}^{\mathrm{NS},(3),0}(N)$, and ${\hat{a}}_{qg,Q}^{(3),0}(N)$, Eqs. (9), (20), (24), (28), (30), to the fixed moments computed in Ref. [10] and found agreement.

The OMEs $A_{qq,Q}^{\mathrm{PS},(3)}(N)$ and $A_{qg,Q}^{\left(3\right)}(N)$ receive contributions $\propto n_{f}T_{F}^{2}C_{A,F}$ only. Due to this we present as well the constant parts of the renormalized OMEs. They read:

(40) $$a_{qq,Q}^{\mathrm{PS},(3),0}=n_{f}T_{F}^{2}C_{F}\left\{\frac{{\left(N^{2}+N+2\right)}^{2}}{\left(N-1)N^{2}{\left(1+N\right)}^{2}(2+N\right)}\left[\frac{80}{27}\left(S_{1}^{3}+3S_{1}S_{2}+2S_{3}\right)+\frac{256}{9}\zeta_{3}\right]-\frac{16R_{1}(N)}{27(N-1)N^{3}{\left(1+N\right)}^{3}{\left(2+N\right)}^{2}}\left[S_{1}^{2}+S_{2}\right]+\frac{32R_{2}(N)}{81(N-1)N^{4}{\left(1+N\right)}^{4}{\left(2+N\right)}^{3}}S_{1}+\frac{R_{3}(N)}{243(N-1)N^{5}{\left(1+N\right)}^{5}{\left(2+N\right)}^{4}}\right\}$$

with

(41) $$R_{1}(N)=40N^{7}+185N^{6}+430N^{5}+521N^{4}+452N^{3}+404N^{2}-16N-96,$$

(42) $$R_{2}(N)=233N^{10}+1744N^{9}+5937N^{8}+11454N^{7}+14606N^{6}+15396N^{5}+12030N^{4}+3272N^{3}-928N^{2}-96N+288,$$

(43) $$R_{3}(N)=-42560N^{13}-445792N^{12}-2124448N^{11}-6005792N^{10}-11345024N^{9}-15758592N^{8}-17045248N^{7}-13567040N^{6}-6545312N^{5}-1096768N^{4}+374528N^{3}+109056N^{2}+32256N+27648,$$

and

(44) $$a_{qg,Q}^{(3),0}=n_{f}T_{F}^{2}\left\{C_{F}\left[\frac{N^{2}+N+2}{N(N+1)(N+2)}\left[-\frac{56}{9}S_{4}+\frac{32}{27}S_{3}S_{1}+\frac{8}{9}S_{2}S_{1}^{2}+\frac{4}{9}S_{2}^{2}+\frac{4}{27}S_{1}^{4}+\frac{256}{9}S_{1}\zeta_{3}\right]-\frac{16(10N^{3}+13N^{2}+29N+6)}{81N^{2}(1+N)(2+N)}\left[S_{1}^{3}+3S_{2}S_{1}\right]+\frac{32(5N^{3}-16N^{2}+N-6)}{81N^{2}(1+N)(2+N)}S_{3}+\frac{8(109N^{4}+291N^{3}+478N^{2}+324N+40)}{27N^{2}{\left(1+N\right)}^{2}(2+N)}S_{2}+\frac{8(215N^{4}+481N^{3}+930N^{2}+748N+120)}{81N^{2}{\left(1+N\right)}^{2}(2+N)}S_{1}^{2}-\frac{R_{4}(N)}{243N^{2}{\left(1+N\right)}^{3}(2+N)}S_{1}-\frac{64(N^{2}+N+2)R_{5}(N)}{9(N-1)N^{3}{\left(1+N\right)}^{3}{\left(2+N\right)}^{2}}\zeta_{3}+\frac{R_{6}(N)}{243(N-1)N^{6}{\left(1+N\right)}^{6}{\left(2+N\right)}^{5}}\right]+C_{A}\left[\frac{N^{2}+N+2}{N(N+1)(N+2)}\left[-\frac{56}{9}S_{4}-\frac{128}{9}S_{-4}+\frac{160}{27}S_{3}S_{1}-\frac{4}{9}S_{2}^{2}+\frac{8}{9}S_{2}S_{1}^{2}-\frac{4}{27}S_{1}^{4}-\frac{64}{9}S_{2,1}S_{1}-\frac{128}{9}S_{3,1}+\frac{64}{9}S_{2,1,1}-\frac{256}{9}\zeta_{3}S_{1}\right]+\frac{32(5N^{4}+20N^{3}+41N^{2}+49N+20)}{81N{\left(1+N\right)}^{2}{\left(2+N\right)}^{2}}\left[S_{1}^{3}+12S_{2,1}-3S_{2}S_{1}\right]+\frac{64}{81}\frac{\left(5N^{4}+38N^{3}+59N^{2}+31N+20\right)}{N{\left(1+N\right)}^{2}{\left(2+N\right)}^{2}}S_{3}+\frac{128}{27}\frac{\left(5N^{2}+8N+10\right)}{N(1+N)(2+N)}S_{-3}+\frac{512}{9}\frac{\left(N^{2}+N+1)(N^{2}+N+2\right)}{(N-1)N^{2}{\left(1+N\right)}^{2}{\left(2+N\right)}^{2}}\zeta_{3}-\frac{16R_{7}(N)}{81N{\left(1+N\right)}^{3}{\left(2+N\right)}^{3}}S_{2}-\frac{32(121N^{3}+293N^{2}+414N+224)}{81N{\left(1+N\right)}^{2}(2+N)}S_{-2}-\frac{R_{8}(N)}{81N{\left(1+N\right)}^{3}{\left(2+N\right)}^{3}}S_{1}^{2}+\frac{16R_{9}(N)}{243(N-1)N^{2}{\left(1+N\right)}^{4}{\left(2+N\right)}^{4}}S_{1}+\frac{8R_{10}(N)}{243(N-1)N^{5}{\left(1+N\right)}^{5}{\left(2+N\right)}^{5}}\right]\right\},$$

with

(45) $$R_{4}(N)=24368N^{5}+81984N^{4}+179200N^{3}+225232N^{2}+126880N+21504,$$

(46) $$R_{5}(N)=3N^{6}+9N^{5}-N^{4}-17N^{3}-38N^{2}-28N-24,$$

(47) $$R_{6}(N)=13923N^{17}+180999N^{16}+1064857N^{15}+3812487N^{14}+9348807N^{13}+16391845N^{12}+20248499N^{11}+17070917N^{10}+11536274N^{9}+11303496N^{8}+13846104N^{7}+16104128N^{6}+22643488N^{5}+29337472N^{4}+26395008N^{3}+15388416N^{2}+5612544N+995328,$$

(48) $$R_{7}(N)=139N^{6}+1093N^{5}+3438N^{4}+5776N^{3}+5724N^{2}+3220N+752,$$

(49) $$R_{8}(N)=1648N^{6}+11104N^{5}+34368N^{4}+63856N^{3}+71904N^{2}+43264N+10880,$$

(50) $$R_{9}(N)=+1244N^{10}+10557N^{9}+40547N^{8}+90323N^{7}+114495N^{6}+49344N^{5}-69902N^{4}-115200N^{3}-64352N^{2}-11264N+864,$$

(51) $$R_{10}(N)=3315N^{15}+39780N^{14}+194011N^{13}+471164N^{12}+416251N^{11}-860568N^{10}-3525799N^{9}-6015120N^{8}-6333994N^{7}-4373672N^{6}-1907512N^{5}-499824N^{4}-217952N^{3}-264192N^{2}-160128N-34560.$$

In both the constant terms of the renormalized OMEs Eqs. (40), (44) $\zeta_{2}$ does not contribute anymore. Phenomenological applications of the corresponding massive Wilson coefficients are given in Ref. [17].

### 3.2 The operator matrix elements for transversity

Transversity is a twist-2 flavor non-singlet operator matrix element related to a tensor operator, which cannot be accessed in deep-inelastic scattering, but via polarized semi-inclusive deep-inelastic scattering and the polarized Drell–Yan process. The transversity distribution

(52) $$\Delta_{T}f\left(x,Q^{2}\right)\equiv f^{↑}\left(x,Q^{2}\right)-f^{↓}\left(x,Q^{2}\right)$$

contributes to a large variety of scattering processes, cf. [42]. Here ↑(↓) denote the transverse spin directions. Eq. (52) describes the transversity distribution obtained in the light-cone expansion at twist 2 or in the collinear parton model. For other phenomenological applications one may introduce $k_{⊥}$-effects for this distribution, [42]. This, however, has consequences for the twist expansion and the renormalization of the corresponding processes, when calculating them to higher orders. We will therefore restrict the analysis to the level of twist 2 and consider only processes which are free of $k_{⊥}$-effects, or after these were integrated out in the final state.

For semi-inclusive deeply inelastic charged lepton-nucleon scattering $lN\to l^{\prime }h+X$ the Born cross section, after the $P_{h⊥}$-integration, is given by, [42],

(53) $$\frac{d^{3}\sigma }{dx\;dy\;dz}=\frac{4\pi \alpha_{\mathrm{em}}^{2}s}{Q^{4}}\sum_{a=q,\bar{q}}e_{a}^{2}x\left\{\frac{1}{2}\left[1+{\left(1-y\right)}^{2}\right]F_{a}\left(x,Q^{2}\right){\tilde{D}}_{a}\left(z,Q^{2}\right)-(1-y)|S_{⊥}||S_{h⊥}|\cos(\phi_{S}+\phi_{S_{h}})\Delta_{T}F_{a}\left(x,Q^{2}\right)\Delta_{T}{\tilde{D}}_{a}\left(z,Q^{2}\right)\right\}.$$

Here, in addition to the Bjorken variables *x* and *y*, the fragmentation variable *z* occurs. $S_{⊥}$ and $S_{h⊥}$ are the transverse spin vectors of the incoming nucleon *N* and the measured hadron *h*. The angles $\phi_{S,S_{h}}$ are measured in the plane perpendicular to the $\gamma^{⁎}N$ (*z*-)axis between the *x*-axis and the respective vector. The transversity distribution can be obtained from Eq. (53) for a transversely polarized hadron *h* by measuring its polarization. The functions $F_{i},{\tilde{D}}_{i},\Delta_{T}F_{i},\Delta_{T}{\tilde{D}}_{i}$ are given by

(54) $$F_{i}\left(x,Q^{2}\right)=C_{i}\left(x,Q^{2}\right)⊗f_{i}\left(x,Q^{2}\right),$$

(55) $${\tilde{D}}_{i}\left(z,Q^{2}\right)={\tilde{C}}_{i}\left(z,Q^{2}\right)⊗D_{i}\left(z,Q^{2}\right),$$

(56) $$\Delta_{T}F_{i}\left(x,Q^{2}\right)=\Delta_{T}C_{i}\left(x,Q^{2}\right)⊗\Delta_{T}f_{i}\left(x,Q^{2}\right),$$

(57) $$\Delta_{T}{\tilde{D}}_{i}\left(z,Q^{2}\right)=\Delta_{T}{\tilde{C}}_{i}\left(z,Q^{2}\right)⊗\Delta_{T}D_{i}\left(z,Q^{2}\right).$$

Here, ⊗ denotes the Mellin convolution, $D_{i},\Delta_{T}D_{i}$ are the fragmentation functions and $C_{i},\;{\tilde{C}}_{i},\;\Delta_{T}C_{i},\;\Delta_{T}{\tilde{C}}_{i}$ are the corresponding space- and time-like Wilson coefficients. The Wilson coefficient for transversity, $\Delta_{T}C_{i}(x,Q^{2})$, contains light ($\Delta_{T}C_{i}$) and heavy flavor ($\Delta_{T}H_{i}$) contributions

(58) $$\Delta_{T}C_{i}\left(x,Q^{2}\right)=\Delta_{T}C_{i}\left(x,Q^{2}\right)+\Delta_{T}H_{i}\left(x,Q^{2}\right).$$

For brevity we dropped arguments like $m^{2}$, the factorization scale, $\mu^{2}$, and the number of light flavors, $N_{f}$, in Eq. (58).

Eq. (53) holds for spin-1/2 hadrons in the final state, but the transversity distribution may also be measured in the lepto-production process of spin-1 hadrons, [43]. In this case, the $P_{h⊥}$-integrated Born cross section reads

(59) $$\frac{d^{3}\sigma }{dx\;dy\;dz}=\frac{4\pi \alpha^{2}}{xyQ^{2}}\sin(\phi_{S}+\phi_{S_{LT}})|S_{⊥}||S_{LT}|(1-y)\sum_{i=q,\bar{q}}e_{i}^{2}x\Delta_{T}F_{i}\left(x,Q^{2}\right){\hat{H}}_{i,1,LT}\left(z,Q^{2}\right).$$

Here, the polarization state of a spin-1 particle is described by a tensor with five independent components, [44]. $\phi_{LT}$ denotes the azimuthal angle of ${\vec{S}}_{LT}$, with

(60) $$|S_{LT}|=\sqrt{{\left(S_{LT}^{x}\right)}^{2}+{\left(S_{LT}^{y}\right)}^{2}}.$$

${\hat{H}}_{a,1,LT}(z,Q^{2})$ is a *T*- and chirally odd twist-2 fragmentation function at vanishing $k_{⊥}$. Process (59) has the advantage that the transverse polarization of the produced hadron can be measured from its decay products.

The transversity distribution can also be measured in the transversely polarized Drell–Yan process using the polarization asymmetry, see Refs. [45], [46], [47]. However, the SIDIS processes have the advantage that in high luminosity experiments, the heavy flavor contributions can be tagged like in deep-inelastic scattering. This is not the case for the Drell–Yan process, where the heavy flavor effects appear as inclusive radiative corrections in the Wilson coefficients.

As was shown in Ref. [8], in the region $Q^{2}\gg m^{2}$ all non-power contributions to the heavy quark Wilson coefficients obey factorization relations. In the general flavor non-singlet case one obtains for $N_{f}$ light and one heavy quark

(61) $$H_{a}^{\text{asymp},\mathrm{NS}}\left(x,\frac{Q^{2}}{m^{2}},\frac{m^{2}}{\mu^{2}}\right)=C_{a,q}^{\mathrm{NS}}\left(x,\frac{Q^{2}}{\mu^{2}},N_{f}+1\right)⊗A_{qq,Q}^{\mathrm{NS}}\left(x,\frac{m^{2}}{\mu^{2}}\right)-C_{a,q}^{\mathrm{NS}}\left(x,\frac{Q^{2}}{\mu^{2}},N_{f}\right),$$

where $C_{a,q}^{\mathrm{NS}}$ is a light flavor Wilson coefficient and $A_{qq,Q}^{\mathrm{NS}}$ is the corresponding massive operator matrix element, cf. [10].

The anomalous dimensions for transversity are known to NLO [48] and for a series of moments to 3-loop order [34]. The moments N=1…13 of the 3-loop massive OME were calculated in [11]. Similar to the flavor non-singlet massive OME in the vector case we computed the $O(n_{f})$ contributions for the transversity operator. The constant part of the unrenormalized 3-loop OME is given by

(62) $${\hat{a}}_{qq,Q}^{\mathrm{TR},(3),0}=n_{f}T_{F}^{2}C_{F}\left\{\frac{64}{27}S_{4}+\frac{448}{27}\zeta_{3}S_{1}+\frac{32}{9}\zeta_{2}S_{2}-\frac{320}{81}S_{3}-\frac{160}{27}\zeta_{2}S_{1}-\frac{112}{9}\zeta_{3}+\frac{640}{27}S_{2}+\frac{4}{9}\zeta_{2}-\frac{55552}{729}S_{1}+\frac{2(3917N^{4}+7834N^{3}+4157N^{2}-48N-144)}{243N^{2}{\left(1+N\right)}^{2}}\right\}.$$

The expression for general values of *N* agrees with the corresponding contributions to the moments calculated in [11] before. It is interesting to note that for this color factor the vector and tensor operators (28), (62) lead to the same structures in the harmonic sums as for ${\hat{a}}_{qq,Q}^{\mathrm{NS},(3),0}$.

### 3.3 The mathematical structure of the operator matrix elements

The $n_{f}T_{F}^{2}C_{F,A}$-contributions at $O(a_{s}^{3})$ to the massive operator matrix elements contain nested harmonic sums up to weight w=4. This also applies to all individual Feynman diagrams, cf. [49]. In intermediary results, generalizations of harmonic sums occur, see Appendix A. As has been observed in the computation of various other physical quantities before, such as anomalous dimensions and massless Wilson coefficients to 3-loop order [9], [19], [50], unpolarized and polarized massive OMEs to 2-loop order [15], the polarized and unpolarized Drell–Yan and Higgs-boson production cross section, time-like Wilson coefficients, and virtual and soft corrections to Bhabha-scattering [51], the classes of contributing harmonic sums are always the same. They depend on the loop-order and the topologies of Feynman diagrams involved.

In the present case the following harmonic sums emerge:

(63) $$S_{1}$$

$$S_{2},\;S_{-2}$$

$$S_{3},\;S_{-3},\;S_{2,1},\;S_{-2,1}$$

$$S_{4},\;S_{-4},\;S_{3,1},\;S_{-3,1},\;S_{-2,2},\;S_{2,1,1},\;S_{-2,1,1}.$$

Note that this class, as for the other processes mentioned above, does not contain the index {−1}. Moreover, we used the algebraic relations between the harmonic sums, cf. [41]. Furthermore, structural relations exist between harmonic sums, cf. [23], [52], which reduce the set (63) further. Here the sums

(64) $$S_{-2,2},\;S_{3,1}$$

are connected by differential relations w.r.t. their argument *N* to other sums of (63). This is also the case for all single harmonic sums $S_{\pm n}$, n∈N, n>1, using both the differentiation and argument-duplication relation, cf. [29]. Due to this $S_{1}$ represents the class of all single harmonic sums. I.e. only the *six* basic harmonic sums

(65) $$S_{1}$$

$$S_{2,1},\;S_{-2,1}$$

$$S_{-3,1},\;S_{2,1,1},\;S_{-2,1,1}$$

are needed to represent the 3-loop results for the $n_{f}T_{F}^{2}C_{F,A}$-contributions to the OMEs calculated in the present paper. In the final representation we refer to the algebraic basis (63) and consider the basis (65) for a later numerical implementation. We sorted the respective expressions keeping a rational function in *N* in front of the harmonic sums (63) and *ζ*-values, like $\zeta_{2}$ and $\zeta_{3}$.

The harmonic sums emerge from the series-expansion of hypergeometric structures like the Euler *B*- and Γ-functions and the Pochhammer-symbols in the (generalized) hypergeometric functions FQP(ai(ε),bi(ε);1) in the dimensional parameter *ε*. This leads to single harmonic sums first, which, through summation, turn into (multiple) zeta values [40] and nested harmonic sums [29], [30]. The principle steps on the way from single-scale Feynman diagrams to these structures have been described in Ref. [23].

For phenomenological applications the heavy flavor corrections to the structure functions have to be known in *x*-space. Both the evolution of the parton densities and the Wilson coefficients have to be computed at complex values of *N*. The Mellin-inversion is then performed by a numerical contour integral around the singularities of the problem [53]. The analytic continuation of the harmonic sums to complex values of *N* is outlined in Refs. [23], [52], [54].

### 3.4 The OMEs in the small and large *x* region

In the small *x* limit the following leading behaviour of the ${\hat{a}}_{ij}^{(3),0}$, $a_{qq,Q}^{\mathrm{PS},(3),0}$ and $a_{qg,Q}^{(3),0}$ is obtained:

(66) $${\hat{a}}_{Qg}^{(3),0}\propto n_{f}T_{F}^{2}\left\{C_{A}\left[-\frac{18400}{729}+\frac{448}{27}\zeta_{3}+\frac{16}{9}\zeta_{2}\right]+C_{F}\left[-\frac{185408}{729}+\frac{896}{27}\zeta_{3}-\frac{736}{27}\zeta_{2}\right]\right\}\frac{1}{x},$$

(67) $${\hat{a}}_{Qq}^{\mathrm{PS},(3),0}\propto -n_{f}T_{F}^{2}C_{F}\left[-\frac{111104}{729}+\frac{896}{27}\zeta_{3}-\frac{320}{27}\zeta_{2}\right]\frac{1}{x},$$

(68) $${\hat{a}}_{qq,Q}^{\mathrm{PS},(3),0}\propto -n_{f}T_{F}^{2}C_{F}\left[-\frac{111104}{729}+\frac{896}{27}\zeta_{3}-\frac{320}{27}\zeta_{2}\right]\frac{1}{x},$$

(69) $$a_{qq,Q}^{\mathrm{PS},(3),0}\propto n_{f}T_{F}^{2}C_{F}\frac{1024}{27}\left[-\frac{47}{27}+\zeta_{3}\right]\frac{1}{x},$$

(70) $${\hat{a}}_{qq,Q}^{\mathrm{NS},(3),0}\propto -n_{f}T_{F}^{2}C_{F}\frac{16}{81}\ln^{3}\left(\frac{1}{x}\right),$$

(71) $${\hat{a}}_{qg,Q}^{(3),0}\propto n_{f}T_{F}^{2}\left\{C_{A}\left[-\frac{145408}{729}+\frac{448}{27}\zeta_{3}-\frac{64}{3}\zeta_{2}\right]+C_{F}\left[\frac{68608}{729}+\frac{896}{27}\zeta_{3}+\frac{512}{27}\zeta_{2}\right]\right\}\frac{1}{x},$$

(72) $$a_{qg,Q}^{(3),0}\propto n_{f}T_{F}^{2}\left\{C_{A}\left[-\frac{69472}{729}+\frac{512}{27}\zeta_{3}\right]+C_{F}\left[\frac{42688}{729}+\frac{1024}{27}\zeta_{3}\right]\right\}\frac{1}{x},$$

(73) $${\hat{a}}_{qq,Q}^{\mathrm{TR},(3),0}\propto -n_{f}T_{F}^{2}C_{F}\frac{32}{27}\ln\left(\frac{1}{x}\right).$$

In case of the singlet and pure-singlet terms the leading behaviour is ∝1/x, while in the non-singlet cases it is logarithmic. The small-*x* asymptotics of ${\hat{a}}_{Qq}^{\mathrm{PS},(3),0}$ and ${\hat{a}}_{qq,Q}^{\mathrm{PS},(3),0}$ turn out to be the same. The matrix elements are less singular than the leading terms in the massless Wilson coefficients, cf. [9], [55], for which the full contribution behaves $\propto \ln^{5}(x)$ and ∝ln(x)/x in the non-singlet and pure-singlet (gluon) cases, respectively, while for the $n_{f}^{2}$ contributions the same singular behaviour is obtained.

In the large *x* limit one obtains the following leading behaviour,

(74) $${\hat{a}}_{Qg}^{(3),0}\propto n_{f}T_{F}^{2}(C_{A}-C_{F})\frac{32}{27}\ln^{4}(1-x),$$

(75) $${\hat{a}}_{qq,Q}^{\mathrm{NS},(3),0}\propto n_{f}T_{F}^{2}C_{F}\left[\frac{55552}{729}-\frac{448}{27}\zeta_{3}+\frac{160}{27}\zeta_{2}\right]\frac{1}{{\left(1-x\right)}_{+}},$$

(76) $${\hat{a}}_{qg,Q}^{(3),0},a_{qg,Q}^{(3),0}\propto -n_{f}T_{F}^{2}(C_{A}-C_{F})\frac{4}{27}\ln^{4}(1-x),$$

(77) $${\hat{a}}_{qq,Q}^{\mathrm{TR},(3),0}\propto n_{f}T_{F}^{2}C_{F}\left[\frac{55552}{729}-\frac{448}{27}\zeta_{3}+\frac{160}{27}\zeta_{2}\right]\frac{1}{{\left(1-x\right)}_{+}},$$

where

(78) $$\int_{0}^{1}dx\;\frac{1}{{\left(1-x\right)}_{+}}f(x)=\int_{0}^{1}dx\;\frac{f(x)-f(1)}{1-x},$$

and

(79) $${\hat{a}}_{Qq}^{\mathrm{PS},(3),0}\propto -n_{f}T_{F}^{2}C_{F}\frac{16}{27}\frac{S_{1}^{3}}{N^{2}}≃\frac{32}{9}\left[S_{1,3}(x)-\zeta_{4}\right],$$

(80) $${\hat{a}}_{qq,Q}^{\mathrm{PS},(3),0}\propto n_{f}T_{F}^{2}C_{F}\frac{128}{27}\frac{S_{1}^{3}}{N^{2}}≃-\frac{256}{9}\left[S_{1,3}(x)-\zeta_{4}\right],$$

(81) $$a_{qq,Q}^{\mathrm{PS},(3),0}\propto n_{f}T_{F}^{2}C_{F}\frac{80}{27}\frac{S_{1}^{3}}{N^{2}}≃-\frac{160}{9}\left[S_{1,3}(x)-\zeta_{4}\right],$$

cf. [29], [56]. In the latter case regular values are obtained for x→1, where $S_{1,3}(x)$ denotes a Nielsen integral [57],

(82) $$S_{n,p}(x)={\left(-1\right)}^{n+p-1}\frac{1}{(n-1)!p!}\int_{0}^{1}\frac{dz}{z}\ln^{\left(n-1\right)}(z)\ln^{p}(1-xz).$$

The large *x* limits for ${\hat{a}}_{Qg}^{(3),0}$ and ${\hat{a}}_{qg,Q}^{(3),0}$, resp. ${\hat{a}}_{Qq}^{\mathrm{PS},(3),0}$ and ${\hat{a}}_{qq,Q}^{\mathrm{PS},(3),0}$ in the $n_{f}$ term differ by a factor of −8 and −1/8, while the contributions to ${\hat{a}}_{qq,Q}^{\mathrm{NS},(3),0}$ and ${\hat{a}}_{qq,Q}^{\mathrm{TR},(3),0}$ are the same. All terms are less singular compared to the massless cases [9], for which the complete Wilson coefficients behave $\propto {\left[\ln^{5}(1-x)/(1-x)\right]}_{+},\ln^{4}(1-x),\ln^{5}(1-x)$ in the non-singlet, pure-singlet, and gluon case. For the $n_{f}^{2}$ contributions the most singular terms behave like $\propto {\left[\ln^{3}(1-x)/(1-x)\right]}_{+},\ln^{3}(1-x),\ln^{4}(1-x)$, respectively, which are stronger as well, up to the gluon case.

## 4 The contributions to the anomalous dimensions

The anomalous dimensions appear in the 1/ε term of the unrenormalized OMEs, see Ref. [10]. As all other contributions to this term are known, they can be derived by comparing with the 1/ε terms of the present computation.

### 4.1 Vector operators

From the OMEs ${\hat{A}}_{Qg}^{\left(3\right)}(\varepsilon ,N)$ and ${\hat{A}}_{qg,Q}^{\left(3\right)}(\varepsilon ,N)$ one obtains the contributions $O(n_{f}^{2}T_{F}^{2}C_{A,F})$ to the anomalous dimension:

(83) $$\gamma_{qg}^{\left(2\right)}=\frac{n_{f}^{2}T_{F}^{2}}{\left(N+1)(N+2\right)}\left\{C_{A}\left[\left(N^{2}+N+2\right)\left(\frac{128}{3N}S_{2,1}+\frac{32}{9N}S_{1}^{3}+\frac{128}{3N}S_{-3}+\frac{64}{9N}S_{3}-\frac{32}{3N}S_{2}S_{1}\right)-\frac{128(5N^{2}+8N+10)}{9N}S_{-2}-\frac{64(5N^{4}+26N^{3}+47N^{2}+43N+20)}{9N(N+1)(N+2)}S_{2}-\frac{64(5N^{4}+20N^{3}+41N^{2}+49N+20)}{9N(N+1)(N+2)}S_{1}^{2}+\frac{64P_{1}(N)}{27N{\left(N+1\right)}^{2}{\left(N+2\right)}^{2}}S_{1}+\frac{16P_{2}(N)}{27(N-1)N^{4}{\left(N+1\right)}^{3}{\left(N+2\right)}^{3}}\right]+C_{F}\left[\frac{32}{9}\frac{N^{2}+N+2}{N}\left\{10S_{3}-S_{1}^{3}-3S_{1}S_{2}\right\}+\frac{32(5N^{2}+3N+2)}{3N^{2}}S_{2}+\frac{32(10N^{3}+13N^{2}+29N+6)}{9N^{2}}S_{1}^{2}-\frac{32(47N^{4}+145N^{3}+426N^{2}+412N+120)}{27N^{2}(N+1)}S_{1}+\frac{4P_{3}(N)}{27(N-1)N^{5}{\left(N+1\right)}^{4}{\left(N+2\right)}^{3}}\right]\right\},$$

with

(84) $$P_{1}(N)=19N^{6}+124N^{5}+492N^{4}+1153N^{3}+1362N^{2}+712N+152,$$

(85) $$P_{2}(N)=165N^{12}+1485N^{11}+5194N^{10}+8534N^{9}+3557N^{8}-8899N^{7}-10364N^{6}+6800N^{5}+25896N^{4}+30864N^{3}+19904N^{2}+7296N+1152,$$

(86) $$P_{3}(N)=99N^{14}+990N^{13}+4925N^{12}+17916N^{11}+46649N^{10}+72446N^{9}+32283N^{8}-95592N^{7}-267524N^{6}-479472N^{5}-586928N^{4}-455168N^{3}-269760N^{2}-122112N-27648.$$

The $n_{f}^{2}$-contribution to the pure-singlet anomalous dimension results from ${\hat{A}}_{Qq}^{\mathrm{PS},(3)}(\varepsilon ,N)$ and ${\hat{A}}_{qq,Q}^{\mathrm{PS},(3)}(\varepsilon ,N)$:

(87) $$\gamma_{qq}^{\mathrm{PS},(2)}=\frac{n_{f}^{2}T_{F}^{2}C_{F}}{\left(N-1)N^{2}{\left(N+1\right)}^{2}(N+2\right)}\left\{-\frac{32}{3}{\left(N^{2}+N+2\right)}^{2}\left(S_{1}^{2}+S_{2}\right)+\frac{64}{9}\frac{P_{4}(N)}{N(1+N)(2+N)}S_{1}-\frac{64}{27}\frac{P_{5}(N)}{N^{2}{\left(1+N\right)}^{2}{\left(2+N\right)}^{2}}\right\},$$

(88) $$P_{4}(N)=68N^{5}+37N^{6}+8N^{7}-11N^{4}-86N^{3}-56N^{2}-104N-48,$$

(89) $$P_{5}(N)=+52N^{10}+392N^{9}+1200N^{8}+1353N^{7}-317N^{6}-1689N^{5}-2103N^{4}-2672N^{3}-1496N^{2}-48N+144.$$

Both the $O(n_{f})$ contributions to $\gamma_{qg}^{\left(2\right)}$ and $\gamma_{qq}^{\mathrm{PS},(2)}$ have thus been obtained by two independent new calculations.

The $n_{f}^{2}$-contribution in the flavor non-singlet case is derived from ${\hat{A}}_{qq,Q}^{\mathrm{NS},(3)}(\varepsilon ,N)$:

(90) $$\gamma_{qq}^{\mathrm{NS},(2)}=n_{f}^{2}T_{F}^{2}C_{F}\left\{\frac{128}{9}S_{3}-\frac{640}{27}S_{2}-\frac{128}{27}S_{1}+\frac{8}{27}\frac{P_{6}(N)}{N^{3}{\left(1+N\right)}^{3}}\right\},$$

with

(91) $$P_{6}(N)=51N^{6}+153N^{5}+57N^{4}+35N^{3}+96N^{2}+16N-24.$$

The anomalous dimensions agree with the moments, resp. the general results, in Refs. [10], [19], [58]. Due to the algebraic compactification we obtain a lower number of harmonic sums $S_{\vec{a}}(N)$ if compared to Ref. [19], and agree with [50]. For the flavor non-singlet case the anomalous dimension has been calculated in [18].

### 4.2 Tensor operator

The contribution to the transversity anomalous dimension $\propto n_{f}^{2}$ is obtained from the single pole term of ${\hat{A}}_{qq,Q}^{\mathrm{TR},(3)}$,

(92) $$\gamma_{qq}^{\mathrm{TR},(2)}=n_{f}^{2}T_{F}^{2}C_{F}\left\{\frac{128}{9}S_{3}-\frac{640}{27}S_{2}-\frac{128}{27}S_{1}+\frac{8}{9}\frac{\left(17N^{2}+17N-8\right)}{N(1+N)}\right\}.$$

The results for the anomalous dimensions constitute a first independent check of the result obtained in [11], [34]. Again for this color factor the vector- and tensor operators lead to the same structures in the harmonic sums.

## 5 Conclusions

We calculated the $O(n_{f})$ contributions to the massive operator matrix elements at $O(\alpha_{s}^{3})$ contributing to the heavy flavor Wilson coefficients of the deep-inelastic structure function $F_{2}(x,Q^{2})$ and to transversity in the asymptotic region for general values of the Mellin variable *N* in the $\bar{\mathrm{MS}}$-scheme. Two of the 3-loop OMEs, $A_{qq,Q}^{\mathrm{PS},(3)}$ and $A_{qg,Q}^{\left(3\right)}$, are known completely now. The Feynman diagrams contributing are characterized by one massive and (at least) one massless fermion line, with both bubble- and ladder-topologies. The local operator insertions are linked to two fermion lines and a number of gluon lines. The computation of the Feynman parameter integrals has been performed directly by representing the integrals as nested sums over generalized hypergeometric functions, which result into multiple nested sums over products of hypergeometric expressions and harmonic sums. The sums have been solved by applying modern summation technologies in difference and product fields. Although in intermediary results in part of the calculation generalizations of harmonic sums occurred, the final results can be represented in terms of rational expressions of the Mellin variable *N* and of harmonic sums of maximal weight w=4. The harmonic sums contributing show the same structural pattern as being observed in all other massless 2- and 3-loop calculations. Applying also the structural relations, six harmonic sums span the results. The small- and large *x* behaviour of the constant parts of the OMEs has been investigated. In both cases a less singular behaviour than for the massless Wilson coefficients is observed. The OMEs $A_{qq,Q}^{\mathrm{PS},(3)}$ and $A_{qg,Q}^{\left(3\right)}$, being completed, do not contain the constant $\zeta_{2}$ after renormalization. All results were compared to the fixed moments given in [10]. We mention that the present calculation is technically very different from that of computing fixed moments carried out previously. From the single pole parts in the dimensional parameter *ε* of the unrenormalized OMEs one may derive the respective contributions to the 3-loop anomalous dimensions, which are obtained in the three cases $\gamma_{qq}^{\mathrm{PS},(2)},\gamma_{qg}^{\left(2\right)},\gamma_{qq}^{\mathrm{TR},(2)}$ as a first independent recalculation, using a different method. We confirm the results in the literature, both in the deep-inelastic case and for transversity, Refs. [18], [19], [34].

## Appendix A Examples for sums occurring in the calculation

In the present calculation numerous single – to triple finite and infinite sums of an extension of the hypergeometric type had to be calculated. For these sums, depending on various summation parameters, $n_{i}$, the ratio of the summands, except the part containing harmonic sums,

(A.1) $$\frac{a(\ldots ,n_{i}+1,\ldots )}{a(\ldots ,n_{i},\ldots )},\;\forall i$$

is a rational function in all variables $n_{i}$. Sums of this type can be represented by basic sums of a certain type, which are transcendental to each other and form sum- and product-fields, cf. [25] and references therein. The general form of these sums is

(A.2) $$\sum_{k_{1}=1}^{N_{1}(N)}\sum_{k_{2}=1}^{N_{2}(k_{1},N)}\sum_{k_{3}=1}^{N_{3}(k_{1},k_{2},N)}P\left(S_{\vec{a_{1}}}\left({\tilde{s}}_{1}(k_{i},N)\right),\ldots ,S_{\vec{a_{4}}}\left({\tilde{s}}_{4}(k_{i},N)\right)\right)\Gamma \left[\begin{array}{c} s_{1}(k_{i},N),\ldots ,s_{p}(k_{i},N)\\ s_{p+1}(k_{i},N),\ldots ,s_{p+q}(k_{i},N) \end{array}\right],$$

with $P(x_{1},x_{2},x_{3},x_{4})$ a polynomial from $Q(k_{1},k_{2},k_{3},k_{4},N)[x_{1},x_{2},x_{3},x_{4}]$, with ${\tilde{s}}_{1}(k_{i},N)$, $\ldots ,{\tilde{s}}_{4}(k_{i},N)$ and $s_{1}(k_{i},N),\ldots ,s_{p+q}(k_{i},N)$ for some p,q∈N being integer linear in $k_{1}$, $k_{2}$, $k_{3}$, *N*, with $\vec{a_{l}}$ an index set, and with the upper bounds $N_{1}(N)$, $N_{2}(k_{1},N)$, $N_{3}(k_{1},k_{2})$ being either ∞ or being integer linear in its arguments. The generalized Γ-function, cf. [22], usually includes both Beta-functions and binomials.

In the present calculation one faces more complicated sums than occurring in earlier two-loop calculations up to O(ε), [15]. Partly they may reach higher weight than appearing in the final result. In the following we present a few examples.

(A.3) $$\sum_{j_{1}=1}^{N-2}\sum_{n=1}^{\infty }{\left(-1\right)}^{j_{1}}B(n,N-j_{1})\left(\begin{array}{c} N-2\\ j_{1} \end{array}\right)\frac{S_{2}(-j_{1}+n+N)}{n^{2}(j_{1}-N-2)}=\left\{{\left(-1\right)}^{N}\frac{6-23N+9N^{2}+2N^{3}}{2{\left(N-1\right)}^{2}N^{2}(1+N)(2+N)}+\left[\frac{1}{N+2}-\frac{27{\left(-1\right)}^{N}}{\left(N-1)N(N+1)(N+2\right)}\right]S_{1}-\frac{1}{N(N+2)}\right\}S_{2}^{2}+\left[\frac{1}{N+2}-\frac{48{\left(-1\right)}^{N}}{\left(N-1)N(N+1)(N+2\right)}\right]S_{3}S_{2}-\frac{2S_{-2}^{2}}{N(N+2)}+\left\{-{\left(-1\right)}^{N}\frac{7(12+6N-37N^{2}+6N^{3}+N^{4})}{20{\left(-1+N\right)}^{2}N^{2}(1+N)(2+N)}+\left[-{\left(-1\right)}^{N}\frac{21}{5(-1+N)N(1+N)(2+N)}-\frac{7}{10(N+2)}\right]S_{1}+\frac{7}{10N(N+2)}\right\}\zeta_{2}^{2}+\left\{{\left(-1\right)}^{N}\frac{6-23N+9N^{2}+2N^{3}}{2{\left(-1+N\right)}^{2}N^{2}(1+N)(2+N)}+\left[\frac{3{\left(-1\right)}^{N}}{\left(N-1)N(N+1)(N+2\right)}+\frac{3}{N+2}\right]S_{1}-\frac{3}{N(N+2)}\right\}S_{4}+\left[\frac{3}{N+2}-\frac{18{\left(-1\right)}^{N}}{\left(N-1)N(N+1)(N+2\right)}\right]S_{5}+\left[\frac{2S_{-2}^{2}}{N+2}+\frac{{\left(-1\right)}^{N}(3N-1)}{{\left(N-1\right)}^{3}N^{3}}\right]S_{1}+\frac{2}{2+N}S_{-2}S_{-3}+\left\{-{\left(-1\right)}^{N}\frac{3(12-6N-14N^{2}+7N^{3}+12N^{4}+N^{5})}{{\left(-1+N\right)}^{3}N^{3}(1+N)(2+N)}+{\left(-1\right)}^{N}\frac{9S_{1}^{2}}{\left(-1+N)N(1+N)(2+N\right)}+{\left(-1\right)}^{N}\frac{3(6-23N+9N^{2}+2N^{3})}{{\left(-1+N\right)}^{2}N^{2}(1+N)(2+N)}S_{1}+\left[\frac{3{\left(-1\right)}^{N}}{\left(N-1)N(N+1)(N+2\right)}-\frac{1}{N+2}\right]S_{2}\right\}S_{2,1}+\left[{\left(-1\right)}^{N}\frac{2(12-37N+9N^{2}+4N^{3})}{{\left(-1+N\right)}^{2}N^{2}(1+N)(2+N)}+{\left(-1\right)}^{N}\frac{24}{\left(-1+N)N(1+N)(2+N\right)}S_{1}\right]S_{3,1}+\frac{2S_{3,2}}{N+2}+\left[-\frac{12{\left(-1\right)}^{N}}{\left(N-1)N(N+1)(N+2\right)}-\frac{3}{N+2}\right]S_{4,1}-\frac{2S_{-2}S_{-2,1}}{2+N}+\frac{4S_{-3,-2}}{N+2}+\left[-{\left(-1\right)}^{N}\frac{2(6-23N+9N^{2}+2N^{3})}{{\left(-1+N\right)}^{2}N^{2}(1+N)(2+N)}-{\left(-1\right)}^{N}\frac{12}{\left(-1+N)N(1+N)(2+N\right)}S_{1}\right]S_{2,1,1}-\frac{2S_{-2,1,-2}}{N+2}+{\left(-1\right)}^{N}\frac{1}{\left(-1+N)N(1+N)(2+N\right)}[42S_{2,2,1}-24S_{3,1,1}+54S_{2,1,1,1}]-{\left(-1\right)}^{N}\frac{30}{\left(-1+N)N(1+N)(2+N\right)}S_{3}{\tilde{S}}_{1}\left(\frac{1}{2}\right){\tilde{S}}_{1}(2)+{\left(-1\right)}^{N}\frac{30}{\left(-1+N)N(1+N)(2+N\right)}S_{1}{\tilde{S}}_{1}\left(\frac{1}{2}\right){\tilde{S}}_{3}(2)+\left\{\frac{{\left(-1\right)}^{N}2^{N+2}}{{\left(-1+N\right)}^{3}N}+\left[-{\left(-1\right)}^{N}\frac{2(6-12N+7N^{2}+N^{3})}{{\left(-1+N\right)}^{3}N^{3}}+{\left(-1\right)}^{N}\frac{6S_{1}^{2}}{\left(-1+N)N(1+N)(2+N\right)}+{\left(-1\right)}^{N}\frac{2(6-23N+9N^{2}+2N^{3})}{{\left(-1+N\right)}^{2}N^{2}(1+N)(2+N)}S_{1}\right]{\tilde{S}}_{1}(2)+\left[{\left(-1\right)}^{N}\frac{2(6-23N+9N^{2}+2N^{3})}{{\left(-1+N\right)}^{2}N^{2}(1+N)(2+N)}+{\left(-1\right)}^{N}\frac{12}{\left(-1+N)N(1+N)(2+N\right)}S_{1}\right]{\tilde{S}}_{2}(2)+{\left(-1\right)}^{N}\frac{6}{\left(-1+N)N(1+N)(2+N\right)}{\tilde{S}}_{3}(2)\right\}{\tilde{S}}_{1,1}\left(\frac{1}{2},1\right)+\left\{\left[{\left(-1\right)}^{N}\frac{12S_{1}^{2}}{\left(-1+N)N(1+N)(2+N\right)}+{\left(-1\right)}^{N}\frac{2(6-23N+9N^{2}+2N^{3})}{{\left(-1+N\right)}^{2}N^{2}(1+N)(2+N)}S_{1}\right]{\tilde{S}}_{1}\left(\frac{1}{2}\right)+\left[-{\left(-1\right)}^{N}\frac{2(6-23N+9N^{2}+2N^{3})}{{\left(-1+N\right)}^{2}N^{2}(1+N)(2+N)}-{\left(-1\right)}^{N}\frac{12}{\left(-1+N)N(1+N)(2+N\right)}S_{1}\right]{\tilde{S}}_{1,1}\left(\frac{1}{2},1\right)\right\}{\tilde{S}}_{1,1}(2,1)+\left[-{\left(-1\right)}^{N}\frac{2(6-12N+7N^{2}+N^{3})}{{\left(-1+N\right)}^{3}N^{3}}+{\left(-1\right)}^{N}\frac{6S_{1}^{2}}{\left(-1+N)N(1+N)(2+N\right)}+{\left(-1\right)}^{N}\frac{2(6-23N+9N^{2}+2N^{3})}{{\left(-1+N\right)}^{2}N^{2}(1+N)(2+N)}S_{1}+{\left(-1\right)}^{N}\frac{30}{\left(-1+N)N(1+N)(2+N\right)}S_{2}\right]{\tilde{S}}_{1,2}\left(\frac{1}{2},2\right)-{\left(-1\right)}^{N}\frac{6}{\left(-1+N)N(1+N)(2+N\right)}{\tilde{S}}_{1,1}\left(\frac{1}{2},1\right){\tilde{S}}_{1,2}(2,1)+\left[{\left(-1\right)}^{N}\frac{2(6-23N+9N^{2}+2N^{3})}{{\left(-1+N\right)}^{2}N^{2}(1+N)(2+N)}+{\left(-1\right)}^{N}\frac{12}{\left(-1+N)N(1+N)(2+N\right)}S_{1}\right]\left[{\tilde{S}}_{1,3}\left(\frac{1}{2},2\right)-{\tilde{S}}_{1,3}\left(2,\frac{1}{2}\right)\right]+\frac{{\left(-1\right)}^{N}}{\left(-1+N)N(1+N)(2+N\right)}\left\{30{\tilde{S}}_{1}\left(\frac{1}{2}\right){\tilde{S}}_{1,3}(2,1)+36{\tilde{S}}_{1,4}\left(\frac{1}{2},2\right)+\left[-30S_{1}{\tilde{S}}_{1}\left(\frac{1}{2}\right)-30{\tilde{S}}_{2}\left(\frac{1}{2}\right)+30{\tilde{S}}_{1,1}\left(\frac{1}{2},1\right)\right]{\tilde{S}}_{2,1}(1,2)\right\}+\left\{\left[{\left(-1\right)}^{N}\frac{2(6-23N+9N^{2}+2N^{3})}{{\left(-1+N\right)}^{2}N^{2}(1+N)(2+N)}+{\left(-1\right)}^{N}\frac{24}{\left(-1+N)N(1+N)(2+N\right)}S_{1}\right]{\tilde{S}}_{1}\left(\frac{1}{2}\right)\right\}{\tilde{S}}_{2,1}(2,1)+\frac{{\left(-1\right)}^{N}}{\left(-1+N)N(1+N)(2+N\right)}\left[-12{\tilde{S}}_{1,1}\left(\frac{1}{2},1\right){\tilde{S}}_{2,1}(2,1)+30{\tilde{S}}_{2,3}\left(\frac{1}{2},2\right)-12{\tilde{S}}_{2,3}\left(2,\frac{1}{2}\right)-18{\tilde{S}}_{1}\left(\frac{1}{2}\right){\tilde{S}}_{3,1}(2,1)+30{\tilde{S}}_{3,2}\left(\frac{1}{2},2\right)-30{\tilde{S}}_{4,1}\left(\frac{1}{2},2\right)\right]+\left[{\left(-1\right)}^{N}\frac{2(6-12N+7N^{2}+N^{3})}{{\left(-1+N\right)}^{3}N^{3}}-{\left(-1\right)}^{N}\frac{6S_{1}^{2}}{\left(-1+N)N(1+N)(2+N\right)}-{\left(-1\right)}^{N}\frac{2(6-23N+9N^{2}+2N^{3})}{{\left(-1+N\right)}^{2}N^{2}(1+N)(2+N)}S_{1}-{\left(-1\right)}^{N}\frac{30}{\left(-1+N)N(1+N)(2+N\right)}S_{2}\right]\left[{\tilde{S}}_{1,1,1}\left(\frac{1}{2},1,2\right)+{\tilde{S}}_{1,1,1}\left(\frac{1}{2},2,1\right)\right]+\left[-{\left(-1\right)}^{N}\frac{2(6-23N+9N^{2}+2N^{3})}{{\left(-1+N\right)}^{2}N^{2}(1+N)(2+N)}-{\left(-1\right)}^{N}\frac{12}{\left(-1+N)N(1+N)(2+N\right)}S_{1}\right]{\tilde{S}}_{1}\left(\frac{1}{2}\right){\tilde{S}}_{1,1,1}(1,2,1)+{\left(-1\right)}^{N}\frac{12}{\left(-1+N)N(1+N)(2+N\right)}{\tilde{S}}_{1,1}\left(\frac{1}{2},1\right){\tilde{S}}_{1,1,1}(2,1,1)+\left[-{\left(-1\right)}^{N}\frac{2(6-23N+9N^{2}+2N^{3})}{{\left(-1+N\right)}^{2}N^{2}(1+N)(2+N)}-{\left(-1\right)}^{N}\frac{12}{\left(-1+N)N(1+N)(2+N\right)}S_{1}\right]\left[{\tilde{S}}_{1,1,2}\left(\frac{1}{2},1,2\right)-{\tilde{S}}_{1,1,2}\left(2,\frac{1}{2},1\right)-2{\tilde{S}}_{1,1,2}\left(2,1,\frac{1}{2}\right)\right]-\frac{{\left(-1\right)}^{N}}{\left(-1+N)N(1+N)(2+N\right)}\left[66{\tilde{S}}_{1,1,3}\left(\frac{1}{2},1,2\right)+36{\tilde{S}}_{1,1,3}\left(\frac{1}{2},2,1\right)+30{\tilde{S}}_{1,1,3}\left(1,\frac{1}{2},2\right)+30{\tilde{S}}_{1,1,3}\left(1,2,\frac{1}{2}\right)\right]+\left[-{\left(-1\right)}^{N}\frac{4(6-23N+9N^{2}+2N^{3})}{{\left(-1+N\right)}^{2}N^{2}(1+N)(2+N)}-{\left(-1\right)}^{N}\frac{24}{\left(-1+N)N(1+N)(2+N\right)}S_{1}\right]\left[{\tilde{S}}_{1,2,1}\left(\frac{1}{2},2,1\right)-{\tilde{S}}_{1,2,1}\left(2,\frac{1}{2},1\right)-\frac{1}{2}{\tilde{S}}_{1,2,1}\left(2,1,\frac{1}{2}\right)\right]-{\left(-1\right)}^{N}\frac{12}{\left(-1+N)N(1+N)(2+N\right)}{\tilde{S}}_{1}\left(\frac{1}{2}\right){\tilde{S}}_{1,2,1}(1,2,1)-{\left(-1\right)}^{N}\frac{1}{\left(-1+N)N(1+N)(2+N\right)}\left[30{\tilde{S}}_{1,2,2}\left(\frac{1}{2},1,2\right)+36{\tilde{S}}_{1,2,2}\left(\frac{1}{2},2,1\right)+48{\tilde{S}}_{1,3,1}\left(\frac{1}{2},2,1\right)+30{\tilde{S}}_{1,3,1}\left(1,\frac{1}{2},2\right)+30{\tilde{S}}_{1,3,1}\left(1,2,\frac{1}{2}\right)+30{\tilde{S}}_{2,1,2}\left(\frac{1}{2},2,1\right)+30{\tilde{S}}_{2,1,2}\left(1,\frac{1}{2},2\right)-12{\tilde{S}}_{2,1,2}\left(2,\frac{1}{2},1\right)-24{\tilde{S}}_{2,1,2}\left(2,1,\frac{1}{2}\right)+30{\tilde{S}}_{2,2,1}\left(1,2,\frac{1}{2}\right)-24{\tilde{S}}_{2,2,1}\left(2,\frac{1}{2},1\right)-12{\tilde{S}}_{2,2,1}\left(2,1,\frac{1}{2}\right)+30{\tilde{S}}_{3,1,1}\left(\frac{1}{2},1,2\right)+30{\tilde{S}}_{3,1,1}\left(\frac{1}{2},2,1\right)\right]+\left[{\left(-1\right)}^{N}\frac{2(6-23N+9N^{2}+2N^{3})}{{\left(-1+N\right)}^{2}N^{2}(1+N)(2+N)}+{\left(-1\right)}^{N}\frac{12}{\left(-1+N)N(1+N)(2+N\right)}S_{1}\right]\left[{\tilde{S}}_{1,1,1,1}\left(\frac{1}{2},1,2,1\right)-2{\tilde{S}}_{1,1,1,1}\left(2,\frac{1}{2},1,1\right)-2{\tilde{S}}_{1,1,1,1}\left(2,1,\frac{1}{2},1\right)-2{\tilde{S}}_{1,1,1,1}\left(2,1,1,\frac{1}{2}\right)\right]+\frac{{\left(-1\right)}^{N}}{\left(-1+N)N(1+N)(2+N\right)}\left[66{\tilde{S}}_{1,1,1,2}\left(\frac{1}{2},1,2,1\right)+48{\tilde{S}}_{1,1,1,2}\left(\frac{1}{2},2,1,1\right)+30{\tilde{S}}_{1,1,1,2}\left(1,\frac{1}{2},2,1\right)+30{\tilde{S}}_{1,1,1,2}\left(1,2,\frac{1}{2},1\right)+30{\tilde{S}}_{1,1,2,1}\left(\frac{1}{2},1,1,2\right)+12{\tilde{S}}_{1,1,2,1}\left(\frac{1}{2},1,2,1\right)+48{\tilde{S}}_{1,1,2,1}\left(\frac{1}{2},2,1,1\right)+30{\tilde{S}}_{1,1,2,1}\left(1,\frac{1}{2},1,2\right)+30{\tilde{S}}_{1,1,2,1}\left(1,2,1,\frac{1}{2}\right)+30{\tilde{S}}_{1,2,1,1}\left(\frac{1}{2},1,1,2\right)+30{\tilde{S}}_{1,2,1,1}\left(\frac{1}{2},1,2,1\right)+12{\tilde{S}}_{1,2,1,1}\left(\frac{1}{2},2,1,1\right)+30{\tilde{S}}_{1,2,1,1}\left(1,1,\frac{1}{2},2\right)+30{\tilde{S}}_{1,2,1,1}\left(1,1,2,\frac{1}{2}\right)+30{\tilde{S}}_{2,1,1,1}\left(1,\frac{1}{2},1,2\right)+30{\tilde{S}}_{2,1,1,1}\left(1,\frac{1}{2},2,1\right)+30{\tilde{S}}_{2,1,1,1}\left(1,1,\frac{1}{2},2\right)+30{\tilde{S}}_{2,1,1,1}\left(1,1,2,\frac{1}{2}\right)+30{\tilde{S}}_{2,1,1,1}\left(1,2,\frac{1}{2},1\right)+30{\tilde{S}}_{2,1,1,1}\left(1,2,1,\frac{1}{2}\right)-24{\tilde{S}}_{2,1,1,1}\left(2,\frac{1}{2},1,1\right)-24{\tilde{S}}_{2,1,1,1}\left(2,1,\frac{1}{2},1\right)-24{\tilde{S}}_{2,1,1,1}\left(2,1,1,\frac{1}{2}\right)-12{\tilde{S}}_{1,1,1,1,1}\left(\frac{1}{2},1,2,1,1\right)-36{\tilde{S}}_{1,1,1,1,1}\left(\frac{1}{2},2,1,1,1\right)\right]+\left\{{\left(-1\right)}^{N}\frac{3S_{1}^{2}}{\left(-1+N)N(1+N)(2+N\right)}+{\left(-1\right)}^{N}\frac{2+9N-5N^{2}}{{\left(-1+N\right)}^{2}N(1+N)(2+N)}+{\left(-1\right)}^{N}\frac{6-11N+2N^{2}}{{\left(-1+N\right)}^{2}N^{2}(2+N)}S_{1}+\left[\frac{3{\left(-1\right)}^{N}}{\left(N-1)N(N+1)(N+2\right)}+\frac{1}{N+2}\right]S_{2}\right\}\zeta_{3}+\zeta_{2}\left\{{\left(-1\right)}^{N}\frac{9S_{1}^{2}}{2(-1+N)N(1+N)(2+N)}+{\left(-1\right)}^{N}\frac{2+3N-2^{2+N}N-2N^{2}-3\cdot 2^{1+N}N^{2}+6N^{3}-2^{1+N}N^{3}-3N^{4}}{{\left(-1+N\right)}^{3}N^{2}(1+N)(2+N)}+\left\{{\left(-1\right)}^{N}\frac{-12+N+27N^{2}-4N^{3}}{2{\left(-1+N\right)}^{2}N^{2}(1+N)(2+N)}-\left[\frac{1}{N+2}+\frac{6{\left(-1\right)}^{N}}{\left(N-1)N(N+1)(N+2\right)}\right]S_{1}+\frac{1}{N(N+2)}\right\}S_{2}+\left[-\frac{6{\left(-1\right)}^{N}}{\left(N-1)N(N+1)(N+2\right)}-\frac{2}{N+2}\right]S_{3}-\frac{2S_{-2}}{N(N+2)}+\left[{\left(-1\right)}^{N}\frac{-6+3N+18N^{2}-20N^{3}-3N^{4}+2N^{5}}{{\left(-1+N\right)}^{3}N^{3}(1+N)(2+N)}+\frac{2S_{-2}}{N+2}\right]S_{1}+\frac{3S_{-3}}{N+2}+{\left(-1\right)}^{N}\frac{12}{\left(-1+N)N(1+N)(2+N\right)}S_{2,1}-\frac{2S_{-2,1}}{N+2}+\left[-{\left(-1\right)}^{N}\frac{3S_{1}^{2}}{\left(-1+N)N(1+N)(2+N\right)}+{\left(-1\right)}^{N}\frac{6-12N+7N^{2}+N^{3}}{{\left(-1+N\right)}^{3}N^{3}}+{\left(-1\right)}^{N}\frac{-6+23N-9N^{2}-2N^{3}}{{\left(-1+N\right)}^{2}N^{2}(1+N)(2+N)}S_{1}\right]{\tilde{S}}_{1}(2)+\left[{\left(-1\right)}^{N}\frac{-6+23N-9N^{2}-2N^{3}}{{\left(-1+N\right)}^{2}N^{2}(1+N)(2+N)}-{\left(-1\right)}^{N}\frac{6}{\left(-1+N)N(1+N)(2+N\right)}S_{1}\right]{\tilde{S}}_{2}(2)+\left[{\left(-1\right)}^{N}\frac{6-23N+9N^{2}+2N^{3}}{{\left(-1+N\right)}^{2}N^{2}(1+N)(2+N)}+{\left(-1\right)}^{N}\frac{6}{\left(-1+N)N(1+N)(2+N\right)}S_{1}\right]{\tilde{S}}_{1,1}(2,1)-{\left(-1\right)}^{N}\frac{3}{\left(-1+N)N(1+N)(2+N\right)}{\tilde{S}}_{3}(2)+\frac{{\left(-1\right)}^{N}}{\left(-1+N)N(1+N)(2+N\right)}\left[3{\tilde{S}}_{1,2}(2,1)-15{\tilde{S}}_{2,1}(1,2)+6{\tilde{S}}_{2,1}(2,1)-6{\tilde{S}}_{1,1,1}(2,1,1)\right]+\left[\frac{3}{N+2}-\frac{18{\left(-1\right)}^{N}}{\left(N-1)N(N+1)(N+2\right)}\right]\zeta_{3}\right\}+\left[\frac{27{\left(-1\right)}^{N}}{\left(N-1)N(N+1)(N+2\right)}-\frac{9}{2(N+2)}\right]\zeta_{5}-30{\left(-1\right)}^{N}\frac{1}{\left(N-1)N(N+1)(N+2\right)}{\tilde{S}}_{1}\left(\frac{1}{2}\right){\tilde{S}}_{1,1,2}(1,2,1),$$

with

(A.4) $$B(a,b)=\frac{\Gamma (a)\Gamma (b)}{\Gamma (a+b)}.$$

(A.5) $$\sum_{j=1}^{N-2}\sum_{j_{1}=1}^{-j+N-2}\sum_{n=1}^{\infty }\frac{{\left(-1\right)}^{j_{1}}jB(j,n)\left(\begin{array}{c} -j+N-2\\ j_{1} \end{array}\right)S_{1}(j)S_{1}(n)}{\left(j+n)(j+n+1)(j+n+2)(j+n+3)(j_{1}-N-2\right)}={\left(-1\right)}^{N}\frac{-1+2N-3N^{2}}{N^{3}(1+N)(2+N)}+\frac{-16+12N+10N^{2}-17N^{3}-31N^{4}}{8N^{3}(1+N)(2+N)}+\left[{\left(-1\right)}^{N}\left[-\frac{1}{N(2+N)}S_{1}+\frac{1}{\left(N+1)(N+2\right)}\right]+\frac{1}{4(N+2)}\right]S_{3}-\frac{S_{4}}{2(N+2)}-{\left(-1\right)}^{N}\frac{4}{\left(1+N)(2+N\right)}S_{-2}+S_{2}\left\{\frac{-8-2N+N^{2}+5N^{3}+5N^{4}}{4N^{3}(1+N)(2+N)}+\left[\frac{{\left(-1\right)}^{N}(N-1)}{N^{2}(N+2)}+\frac{1}{2N^{2}(N+1)}\right]S_{1}+{\left(-1\right)}^{N}\frac{2}{N(2+N)}S_{-2}-\frac{2{\left(-1\right)}^{N}}{\left(N+1)(N+2\right)}\right\}+{\left(-1\right)}^{N}\frac{1+2N^{2}}{N^{2}(1+N)(2+N)}S_{-3}+S_{1}\left\{\frac{2+N+N^{2}}{2N^{2}(1+N)(2+N)}+{\left(-1\right)}^{N}\left[\frac{2(-1+N)}{N^{2}(2+N)}S_{-2}-\frac{3}{N(2+N)}S_{-3}\right]\right\}+\frac{3{\left(-1\right)}^{N}}{N(2+N)}S_{-4}+\left[\frac{{\left(-1\right)}^{N}(1-N)}{N^{2}(N+2)}+\frac{1}{2(N+2)}\right]S_{2,1}-{\left(-1\right)}^{N}\frac{2}{N(2+N)}S_{2,-2}+\left[\frac{{\left(-1\right)}^{N}}{N(N+2)}+\frac{1}{2(N+2)}\right]S_{3,1}+\left[{\left(-1\right)}^{N}\frac{2}{N(2+N)}S_{1}-{\left(-1\right)}^{N}\frac{2(-1+2N^{2})}{N^{2}(1+N)(2+N)}\right]S_{-2,1}+{\left(-1\right)}^{N}\left[\frac{2}{N(2+N)}S_{-3,1}-\frac{4}{N(2+N)}S_{-2,1,1}\right]+\left[\frac{16+4N-2N^{2}+N^{3}+N^{4}}{8N^{3}(1+N)(2+N)}-\frac{S_{1}}{2N^{2}(N+1)}+\frac{{\left(-1\right)}^{N}}{N^{3}(N+1)(N+2)}\right]\zeta_{2},$$

(A.6) $$\sum_{j=1}^{N-2}\sum_{j_{1}=1}^{-j+N-2}\sum_{n=1}^{\infty }\frac{{\left(-1\right)}^{j_{1}}jB(j,n)\left(\begin{array}{c} -j+N-2\\ j_{1} \end{array}\right)S_{2}(n)}{\left(j+n)(j+n+1)(j+n+2)(j+n+3)(j_{1}-N-2\right)}=-\frac{S_{2}^{2}}{4(N+2)}+\left[\frac{{\left(-1\right)}^{N}S_{1}^{2}}{2N(N+2)}+\left[-\frac{{\left(-1\right)}^{N}}{N(N+2)}-\frac{1}{2N^{2}(N+1)}\right]S_{1}-\frac{N^{2}+1}{2N^{2}(N+1)}+{\left(-1\right)}^{N}\left[\frac{1}{N(2+N)}S_{-2}+\frac{1}{\left(N+1)(N+2\right)}\right]\right]S_{2}+{\left(-1\right)}^{N}\frac{8-19N+24N^{2}}{8(-1+N)N^{2}(1+N)(2+N)}+\frac{-48-24N+71N^{2}+95N^{3}}{48N^{2}(1+N)(2+N)}-\frac{S_{3}}{2N^{2}(N+1)}+\left[-\frac{{\left(-1\right)}^{N}}{2N(N+2)}-\frac{1}{4(N+2)}\right]S_{4}+{\left(-1\right)}^{N}\left[\frac{2}{\left(1+N)(2+N\right)}S_{-2}+\frac{1}{N(2+N)}S_{1}^{2}S_{-2}+\frac{1}{N(2+N)}S_{-3}+S_{1}\left[-\frac{2}{N(2+N)}S_{-2}-\frac{1}{N(2+N)}S_{-3}\right]-\frac{1}{N(2+N)}S_{-4}\right]+\left[-{\left(-1\right)}^{N}\frac{1}{N(2+N)}S_{1}+\frac{1}{2N^{2}(N+1)}+\frac{{\left(-1\right)}^{N}}{N(N+2)}\right]S_{2,1}+\left[\frac{2{\left(-1\right)}^{N}}{N(N+2)}-{\left(-1\right)}^{N}\frac{2}{N(2+N)}S_{1}\right]S_{-2,1}+{\left(-1\right)}^{N}\left[\frac{1}{N(2+N)}S_{-3,1}+\frac{1}{N(2+N)}S_{2,1,1}+\frac{2}{N(2+N)}S_{-2,1,1}\right]+\left[{\left(-1\right)}^{N}\frac{-2+N-2N^{2}}{2(-1+N)N^{2}(1+N)(2+N)}+\frac{2+N-N^{2}-2N^{3}}{2N^{2}(1+N)(2+N)}+\frac{S_{1}}{2N^{2}(N+1)}+\left[\frac{1}{2(N+2)}-\frac{{\left(-1\right)}^{N}}{N(N+2)}\right]S_{2}-{\left(-1\right)}^{N}\frac{2}{N(2+N)}S_{-2}\right]\zeta_{2}+\left[\frac{-12-6N+N^{2}+N^{3}}{12N^{2}(1+N)(2+N)}+\frac{{\left(-1\right)}^{N}}{\left(N-1)N^{2}(N+1)(N+2\right)}\right]\zeta_{3},$$

(A.7) $$\sum_{j=1}^{N-2}\sum_{j_{1}=1}^{-j+N-2}\frac{{\left(-1\right)}^{j_{1}}\left(\begin{array}{c} -j+N-2\\ j_{1} \end{array}\right)S_{1}(j)S_{2}(-j_{1}+N)}{\left(j+2)(j_{1}-N-2\right)}=\left[\frac{{\left(-1\right)}^{N}}{2(N+1)(N+2)}-\frac{1}{2(N+2)}\right]S_{2}^{2}+\left[\frac{S_{1}^{2}}{2(N+2)}+\left[\frac{-3-3N-N^{2}}{{\left(1+N\right)}^{2}{\left(2+N\right)}^{2}}+{\left(-1\right)}^{N}\frac{-4-5N-3N^{2}-N^{3}}{N{\left(1+N\right)}^{2}{\left(2+N\right)}^{2}}\right]S_{1}+{\left(-1\right)}^{N}\frac{8+28N+37N^{2}-42N^{4}-38N^{5}-14N^{6}-2N^{7}}{2N^{2}{\left(1+N\right)}^{3}{\left(2+N\right)}^{3}}+\frac{8+28N+49N^{2}+39N^{3}+6N^{4}-10N^{5}-6N^{6}-N^{7}}{N^{2}{\left(1+N\right)}^{3}{\left(2+N\right)}^{3}}\right]S_{2}+{\left(-1\right)}^{N}\left[-\frac{2}{\left(1+N)(2+N\right)}S_{-2}S_{2}+\frac{(-8-28N-27N^{2}-8N^{3})S_{1}^{2}}{2N^{2}{\left(1+N\right)}^{3}{\left(2+N\right)}^{3}}\right]+\frac{4+5N+3N^{2}+N^{3}}{N{\left(1+N\right)}^{3}{\left(2+N\right)}^{2}}+{\left(-1\right)}^{N}\frac{6+2N-8N^{2}-6N^{3}-N^{4}}{{\left(1+N\right)}^{3}{\left(2+N\right)}^{3}}+{\left(-1\right)}^{N}\left[\frac{-4-3N+2N^{2}+3N^{3}+N^{4}}{N{\left(1+N\right)}^{2}{\left(2+N\right)}^{2}}+\frac{1}{\left(1+N)(2+N\right)}S_{1}\right]S_{3}+{\left(-1\right)}^{N}\left[\frac{3}{2(1+N)(2+N)}S_{4}-\frac{2(-4+2N^{2}+N^{3})}{N^{2}{\left(2+N\right)}^{2}}S_{-2}+\frac{3(-4-3N+2N^{2}+3N^{3}+N^{4})}{N{\left(1+N\right)}^{2}{\left(2+N\right)}^{2}}S_{-3}\right]+S_{1}\left[{\left(-1\right)}^{N}\frac{16+8N-4N^{2}-N^{3}}{N^{3}{\left(2+N\right)}^{3}}-{\left(-1\right)}^{N}\frac{2(4+5N+3N^{2}+N^{3})}{N{\left(1+N\right)}^{2}{\left(2+N\right)}^{2}}S_{-2}+{\left(-1\right)}^{N}\frac{3}{\left(1+N)(2+N\right)}S_{-3}+\frac{1}{{\left(N+1\right)}^{2}(N+2)}\right]+{\left(-1\right)}^{N}\left[+\frac{2}{\left(1+N)(2+N\right)}S_{-4}+\frac{4}{\left(1+N)(2+N\right)}S_{2,-2}+\left[-\frac{2(-4-3N+2N^{2}+3N^{3}+N^{4})}{N{\left(1+N\right)}^{2}{\left(2+N\right)}^{2}}-\frac{2}{\left(1+N)(2+N\right)}S_{1}\right]S_{-2,1}-\frac{6}{\left(1+N)(2+N\right)}S_{-3,1}+\frac{4}{\left(1+N)(2+N\right)}S_{-2,1,1}\right]$$

In the above examples also so-called generalized harmonic sums occur [31], [32]. They obey the following recursive definition:

(A.8) $${\tilde{S}}_{m_{1},\ldots }(x_{1},\ldots ;N)=\sum_{i_{1}=1}^{N}\frac{x_{1}^{i_{1}}}{i_{1}^{m_{1}}}\sum_{i_{2}=1}^{i_{1}-1}\frac{x_{2}^{i_{2}}}{i_{2}^{m_{2}}}{\tilde{S}}_{m_{3},\ldots }(x_{3},\ldots ;i_{2})+{\tilde{S}}_{m_{1}+m_{2},m_{3},\ldots }(x_{1}\cdot x_{2},x_{3},\ldots ;N).$$

The sums $\tilde{S}$ may be reduced to nested harmonic sums for $x_{i}\in \{-1,1\}$. In the present calculation the values of $x_{i}$ extend to {−1/2,1/2,−2,2}. These sums occur in ladder like structures, cf. [9], [35], but may also emerge if contributions to 3-loop Feynman diagrams, containing a 2-point insertion, are separated into various terms. They were even observed in case of the more complicated massive 2-loop graphs [15] if large expressions are arbitrarily separated. In part of the sums terms $\propto 2^{N}$, which lead to an exponential growth in the large *N* limit, occur. However, all these contributions cancel for each individual diagram. In the present case the weight of these sums can reach w=5 intermediary, depending on the *ε*-structure of the contribution, although only w=4 sums will emerge in the final results. Examples for these sums are: (A.9)

$${\tilde{S}}_{1}(1/2,N),\;{\tilde{S}}_{2}(-2;N),\;{\tilde{S}}_{2,1}(-1,2;N),\;{\tilde{S}}_{3,1}(-2,-1/2;N),$$

$${\tilde{S}}_{1,1,1,2}(-1,1/2,2,-1;N),\;{\tilde{S}}_{2,3}(-2,-1/2;N),$$

$${\tilde{S}}_{2,2,1}(-1,-1/2,2;N),\text{ etc}.$$

 The algebraic and structural relations for these sums are worked out in Ref. [32]. Similar to the case of harmonic sums, corresponding basis representations are obtained. They allow to simplify involved structures as of Eq. (A.3) and finally lead to the reduction of the results for the individual diagrams to a representation just in terms of nested harmonic sums. The nested sums emerging in this work, which were not given before in Refs. [15] and those being closer related to the structure of harmonic sums [30], are of the type illustrated above. The latter have been calculated using C. Schneider's packages Sigma [25], EvaluateInfiniteSums [59] and J. Ablinger's package HarmonicSums [26].

## Footnotes

1 For higher order corrections to the gluonic contributions in the threshold region, cf. [7].

2 For the corresponding contributions in case of transversity see [11].

3 From the representations obtained in the fixed flavor number scheme, variable flavor number schemes may be defined under specific conditions [10], [13] observing the correct matching scales [21].

4 In Ref. [10] we have kept the gauge parameter for part of the moments and found gauge independence. In the present calculation we have compared the results also on the basis of diagrams with the moments obtained there.
